# Mesoporous Carbons of Well-Organized Structure in the Removal of Dyes from Aqueous Solutions

**DOI:** 10.3390/molecules26082159

**Published:** 2021-04-09

**Authors:** Magdalena Blachnio, Anna Derylo-Marczewska, Szymon Winter, Malgorzata Zienkiewicz-Strzalka

**Affiliations:** Faculty of Chemistry, Maria Curie-Sklodowska University, M. Curie-Sklodowska Sq. 3, 20-031 Lublin, Poland; annad@hektor.umcs.lublin.pl (A.D.-M.); winter1972@wp.pl (S.W.); malgorzata.zienkiewicz@poczta.umcs.lublin.pl (M.Z.-S.)

**Keywords:** activated carbon, dye adsorption, adsorption kinetics, adsorption equilibrium

## Abstract

Mesoporous carbons with differentiated properties were synthesized by using the method of impregnation of mesoporous well-organized silicas. The obtained carbonaceous materials and microporous activated carbon were investigated by applying different methods in order to determine their structural, surface and adsorption properties towards selected dyes from aqueous solutions. In order to verify applicability of adsorbents for removing dyes the equilibrium and kinetic experimental data were measured and analyzed by applying various equations and models. The structural and acid-base properties of the investigated carbons were evaluated by Small-Angle X-ray Scattering (SAXS) technique, adsorption/desorption of nitrogen, potentiometric titration, and Transmission Electron Microscopy (TEM). The results of these techniques are complementary, indicating the type of porosity and structural ordering, e.g., the pore sizes determined from the SAXS data are in good agreement with those obtained from nitrogen sorption data. The SAXS and TEM data confirm the regularity of mesoporous carbon structure. The adsorption experiment, especially kinetic measurements, reveals the utility of mesoporous carbons in dye removing, taking into account not only the adsorption uptake but also the adsorption rate.

## 1. Introduction

Dyes in waters, even at very low concentrations, are undesirable due to their hazardous effects on flora, fauna and human beings. Therefore, the decolorization process of water resources is of great significance. Brighter colours and better resistance of synthetic dyes to environmental factors resulted in almost complete displacement of natural dyes from their common usage. Synthetic dyes are widely applied in the textile, pharmaceutical, food processing, leather tanning, cosmetics, plastics, photographic and paper industry. Being applied they are subjected to chemical and physical processes, which might generate secondary products as burdensome for the environment as dyes themselves. It is estimated that almost 100,000 various dyes are used in industry with over 700,000 tons annually production worldwide [1]. About 10–15% of them are released to the environment during a manufacturing process [2]. A significant problem is that concentration of dyes in waters located near an industrial plant can reach up to 7000 mg/L [3]. Although the most frequently reported level of dyes in effluents is in the range of 10 to 250 mg/L [4,5,6], it turns out that even very small quantities (less than 1 ppm for some dyes) might result in unwanted changes in the water physical and chemical characteristics [7]. Dyes present in waters adsorb on the suspended organic matter forming sediments/sludges or due to their relatively high water-solubility remain dissolved [8]. The latter seem to be particularly hazardous to the aqueous ecosystem. The studies have shown that among dyes there are highly toxic, carcinogenic and mutagenic ones. Furthermore, some colouring substances have been related to dysfunction of the kidney, reproductive system, liver, brain and central nervous system of living organisms [9]. They are also responsible for a decrease of light penetration in waters and restraining of photosynthetic activity of plants. In turn, this results in oxygen deficiency and deterioration of the ecological conditions of water [10,11]. All these make an urge need to focus on specific methods and technologies to purify wastewaters before the final disposal so that their quality can meet international standards and enable the access to pure water for future generations.

Because of complex molecular structure, typical dyes are stable, resistant to light, heat, and oxidizing agents [12] making conventional treatment techniques like: ion exchange [13], membrane filtration [14], irradiation [15,16], electrokinetic coagulation [17], oxidation [18], chemical coagulation-flocculation [19], ozonation [20], biodegradation [21,22,23,24] unsuitable for water purification. Although some of them seem to be effective for dye removal, closer insight into the specific character of the mentioned methods reveals lots of disadvantages. Most important are: sensitivity to particle charge [13], requirement of pre-treatment, risk of membrane fouling, high cost of maintenance [14], requirement of O_2_ [15] or H_2_O_2_, excessive amount of chemical usage [18], problem with generated sludge [17,19] or harmful by-products that subsequently create disposal/treatment problems [20,22], slow rate [21], uncontrolled change of working conditions, production of biosolids that can release of plant nutritious substances and leads to an eutrophication of the aquatic environment [23,24].

As an alternative method, adsorption process in the treatment of dye-contaminated wastewaters can be used due to its simplicity, high efficiency, low costs, ease and flexibility of operation, easy desorption. In recent years, lots of novel adsorbents such as functionalized multiwalled carbon nanotubes [25], graphene oxide/clay nanocomposite [26], doped metal oxide [27] or derived from industrial solid wastes [28], agricultural solid wastes [29,30], biopolymers and their composites [31,32,33], sewage sludge [34], fly ash [35] have been reported in the literature. Although all the materials seem to be very promising, activated carbon is still the most common adsorbent for wastewaters treatment of in industrial plants contaminated with dyes. Superiority of activated carbon is related to its well-developed porosity as well as great affinity for various substances. Currently, the activated carbons with microporous characteristics are most widely applied, however, their utility can be confined to low-molecular substances. Therefore, several methods for the synthesis of mesoporous activated carbons based on a large variety of the natural or synthetic precursors were proposed. The simple method of preparation of mesoporous activated carbons is a direct pyrolytic carbonization, followed by physical activation with gaseous agents (carbon dioxide, water vapour) [36]. To improve the mesoporous structure of the final product the second activation is advisable in a fluidized bed reactor [37]. The synthesis process conditions such as type of raw material, temperature, time and activation agent affect development of the internal structure of adsorbents. Generally, mesoporous carbons have been prepared by means of combination of physical and chemical activation with ZnCl_2_ [38], KOH [39], phosphoric or sulfuric acid [40]. A huge disadvantage of these methods is large consumption of reagents for obtaining adsorbents with a satisfied mesoporous structure. Long-term and large-scale production entails an increase of the costs, a corrosion of the equipment and most importantly the environmental burden. A method based on combining the hydrothermal pre-treatment and chemical activation [41], effectively transforming a carbon precursor seems to be a better solution. In the presented methods the following materials were used as carbon precursors: mustard straw, industrial sludge, walnut and pistachio shell, wood. Recently, a synthesis of magnetic mesoporous activated carbon from rice husk through chemical activation with ZnCl_2_ and magnetite incorporation [42] or activated carbon with outstanding structural characteristics prepared by chemical activation and pyrolysis of sodium carboxymethyl cellulose was proposed [43]. All these mesoporous carbons are characterized by a various size pores distribution, including also micropores.

There is a number of reports on the synthesis of ordered mesoporous carbons (OMCs) with a large surface area and well-defined and uniform pore structure as a moderately new class of efficient adsorbents [44]. Depending on the template, there are two synthesis strategies: hard-templating based (impregnation and etching) and soft-templating based (direct synthesis). Generally, the synthesis is based on an assembly of the organic and inorganic precursors to generate the porous carbon framework. Hard template materials comprise different silica series (MCM, SBA, FDU, MSU-H, HMS), ordered structures of colloid nanoparticles or polymer beads [45]. The direct synthesis of OMCs includes self-assembling of a block copolymer surfactant and a carbon precursor without necessity of template removal. The pore structure of the product is strictly determined by the interactions between the initial components as a driving force of the self-assembly of the soft template. The specific structure of OMCs gives possibility for the post-treatment functionalization with chemical reagents increasing the potential utility in water and wastewater treatment [46]. Furthermore, due to the regular structure OMCs seem to be very useful in the studies on the influence of the adsorbent and adsorbate properties on adsorption phenomena. The strictly defined structural properties of mesoporous adsorbents give the possibility of modelling and analysis of the adsorption process [47]. Furthermore, porosity in the range of mesopores facilitates the regeneration process of spent materials and the further multiple usage. The methods of regeneration most applied comprise wet oxidation, elution with suitable solvent, microwave or thermal regeneration [48]. The regeneration of adsorbents is important taking into account the assumptions of environmental sustainability and economic effectiveness [49,50]. Regarding ordered mesoporous carbons it should be emphasized the multidirectional possibilities of their usage in various industrial applications. This includes: water treatment, air filtration, as catalysts or catalyst supports, as gas storage hosts, in electrochemical energy conversion systems and its storage (ultracapacitors, supercapacitors, battery systems, fuel cells) [45,51,52,53,54]. The authors of this study, meeting the growing need to search for new solutions in the field of adsorption have taken steps to synthesize mesoporous carbon materials as effective adsorbents for pollutants removal from aqueous solutions. These materials can find a potential application in the water and industrial wastewater treatment plants. Superiority of MOCs under currently activated carbons used lies in a fast pollutant diffusion in the internal space of adsorbent and shortening time for reaching adsorption equilibrium. It turns out that in the evaluation of effectiveness of the decolorization process, kinetics seems be a critical factor especially in the large-scale technological or environmental applications. The molecular size of numerous dyes is close to the upper limit of micropores or even exceeds the micropores range [55], so the effective usage of microporous activated carbons is limited. This problem concerns not only dyes but also other large organic molecules such as proteins, enzymes, pharmaceuticals, humic acids, polycyclic aromatic hydrocarbons. Reviewing literature on the subject, it turns out that there is a relatively small number of studies of the systematic analysis of properties of mesoporous carbons with a divergent structure in the anionic and cationic dyes removal from aqueous solutions. For this reason, the investigations in this line are of great importance enabling understanding of the mechanisms of adsorption process as a key method applied in water and wastewater treatment technologies.

In this paper a series of mesoporous carbons based on the hard-templating method was obtained. In the synthesis mesoporous silicas were applied as pore structure templates. Differentiation of the synthesis conditions (type of copolymer, temperature and time of the ageing process, carbon precursor) enabled obtaining the materials with a divergent structure. The physicochemical properties of the mesoporous carbonaceous materials were characterized by using various techniques: adsorption/desorption of nitrogen, potentiometric titration, transmission electron microscopy (TEM), small-angle X-ray scattering (SAXS) and thermal analysis. In order to verify their applicability for removing different types of dyes from aqueous solutions, complex adsorption measurements (equilibrium and kinetics) were performed. The experimental isotherms were interpreted on the basis of the adsorption model on the energetically heterogeneous solids by applying the Generalized Langmuir isotherm equation (GL). The kinetic profiles of adsorption process were described by several equations: the pseudo first-order (PFOE), the pseudo second-order (PSOE), the mixed 1.2-order (MOE) equations as well as the multi-exponential equation (m-exp). The mechanisms of dyes adsorption were studied and correlated with the properties of adsorbates, adsorbents and experimental conditions of the adsorption process. For comparative purposes, the adsorption studies were also carried out for the commercial microporous activated carbon.

## 2. Results and Discussion

### 2.1. Characterization of the Adsorbents

**Figure 1:**
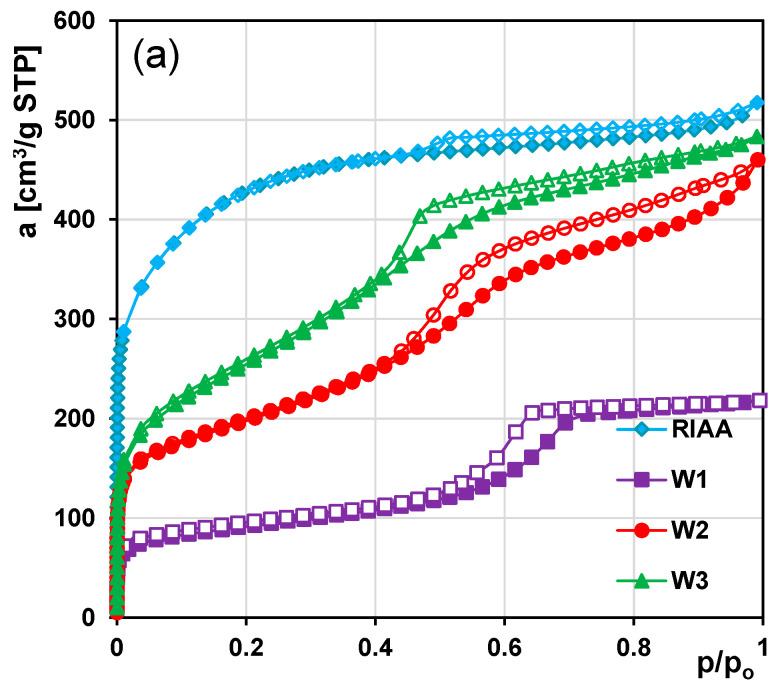

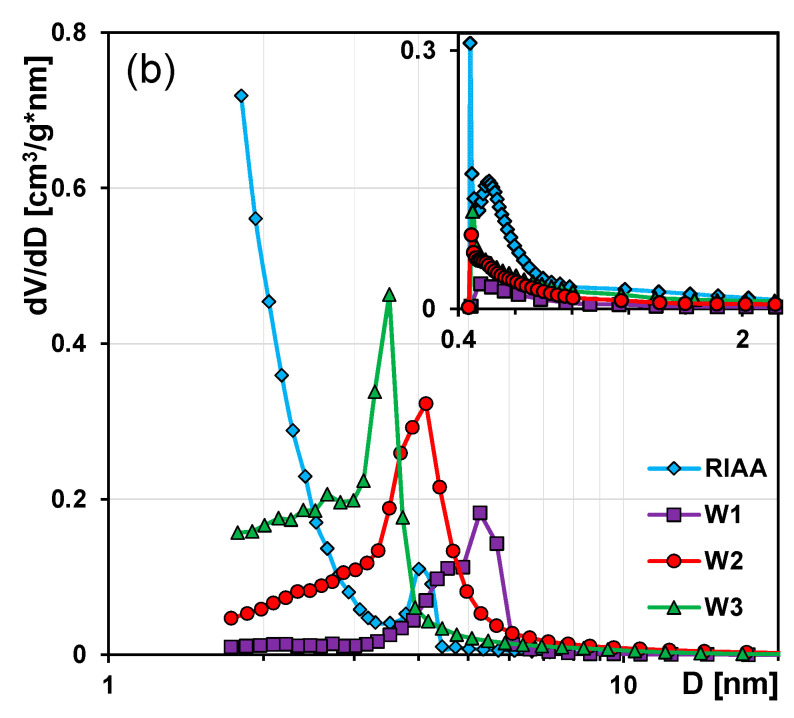
(**a**) The nitrogen adsorption/desorption isotherms for carbonaceous materials. (**b**) Pore size distributions calculated using the Barrett, Joyner, and Halenda BJH and the Horvath–Kawazoe (figure inserted) methods.

In Figure 1 the nitrogen adsorption/desorption isotherms and pore size distributions (PSD) obtained from the desorption branch are presented for the commercial microporous (RIAA) and as-received mesoporous carbons (W1, W2 and W3). For RIAA a steep increase of adsorption in the range of relative pressures lower than 0.05 is observed that proves existence of the well-developed micropore structure (type I according to the IUPAC classification). However, the visible hysteresis loop evidences a partial share of mesopores in the total porosity. In the case of W1, W2 and W3 materials a shape of adsorption isotherms and hysteresis loops at moderate and high relative pressures suggest, the development of porosity in the range of mesopores. The isotherms are typical of mesoporous materials (type IV according to the IUPAC classification). The hysteresis loops for all adsorbents belong to type H4, associated with the presence of slit shaped mesopores. The analysis of PSDs confirms differentiation in the porous structure of the studied adsorbents. For RIAA overwhelming contribution of pores is localized in the micropores range. Additionally, a small peak with max. at 4 nm is also observed. For the series of mesoporous materials, PSDs show relatively sharp peaks suggesting that the porous structure is fairly homogeneous with similar pore sizes. The peak maxima are located in the range of small mesopores—5.3; 4.2 and 3.5 nm for W1, W2 and W3, respectively. The parameters characterizing the textural properties of adsorbents are compared in Table 1. RIAA as a microporous material shows a well-developed pore structure—the highest specific surface area, *S_BET_* and total pore volume, *V_t_* (1468 m^2^/g and 0.80 cm^3^/g, respectively), while among the synthesized carbons, due to a large amount of small mesopores W3 is characterized by significant porosity (*S_BET_* = 908 m^2^/g, *V_t_ =* 0.75 cm^3^/g). The distribution of pore diameters shifted towards higher values and the less developed porous structure (*S_BET_* = 313 m^2^/g, *V_t_ =* 0.34 cm^3^/g) is observed for W1. 

The SAXS effect observed as the scattering intensity I(q) corresponds to the Fourier transform of the square of electron density in the 2-phase system. In the case of the studied systems, the SAXS effect can be applied for carbon and pores as the matrix and scattering phase, respectively, in the small-angle scattering SAXS regime. The SAXS structural parameters determined for the investigated samples are summarized in Table 2.

**Figure 2:**
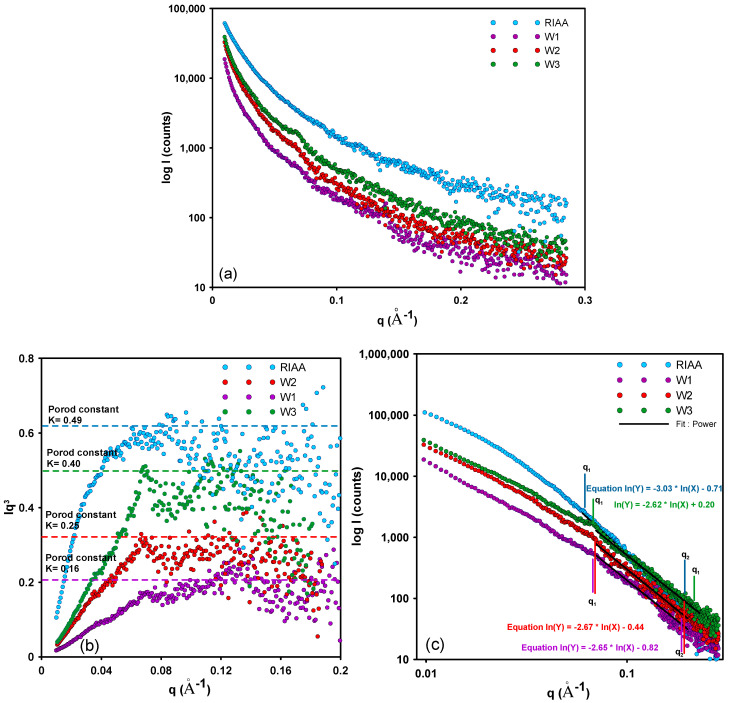
(**a**) Experimental SAXS curves for the investigated materials; (**b**) Porod plots with the slit-collimation calculated for the investigated systems; (**c**) The log-log plots of SAXS intensity in the power-law range for the investigated carbons containing a linear range of Porod area with power approximation.

The structural parameters of activated carbons were determined based on the experimental SAXS curves. Figure 2a shows the experimental SAXS data in the form of continuous curves unbroken in the whole angular measurement range. Typically, the activated carbons are characterized by the random distribution of the scattering heterogeneities (pores) and the absence of correlation in their relative positions, respectively. The microporous carbon RIAA shows a monotonously decaying course of scattering curve in the whole angular measurement range. This suggests the random distribution of the scattering heterogeneities in the carbon structure. However, some repeatability in the pore positions is possible for the templated mesoporous carbons. The scattering curves of the investigated carbons W1-W3 show slight peaks in the scattering vector position of 0.07–0.08 Å^−1^ which suggests the repetitive positions indicating the presence of some structurally ordered forms of pores. The intensity of the characteristic peak was the lowest for the W3 sample which may suggest the weakest level of ordering of all discussed carbons in the mesoporous group (W1-W3). The observed features result from the template procedure of mesoporous carbon synthesis and reflect the colloidal nature of the structure-directing Pluronic copolymer. Despite its presence, the structural order of pores is not maintained to the extent that allows for a clear indication of a specific crystallographic structure. The scattering level (scattering intensity (I(q)) was the highest for the microporous carbon RIAA. The micropores present in a significant amount are a heavily scattering factor.

The scattering level is related to the specific surface of porous systems. The Porod approximation (for the slit collimation system) was observed for all investigated materials as asymptotic behavior of the scattering intensity curves for large q values (Figure 2b). The asymptotic behavior of calculated curves indicates the existence of a two-phase system (carbon matrix and pores) with a sharp boundary. The obtained values of the Porod constants allow to compare the surface areas of the studied materials. The surface area of RIAA carbon is significantly larger than the interface surface of mesoporous carbon. Analyzing the group of materials W1-W3, one can state that the Porod dependencies allow to indicate the highest value for the W3 system and the lowest for W1. Thus, the values of the interfacial surface are consistent with the data obtained by the gas sorption method (*S_BET_* = 1468 m^2^/g, 313 m^2^/g, 679 m^2^/g, and 908 m^2^/g for RIAA, W1, W2, and W3 carbons, respectively). The exact values of *S_SAXS_* were calculated using the SAXS data from the S/V values according to the Dv(R) procedure, and they are presented in Table 2. The interfacial surface of carbon samples equals *S_SAXS_* = 1568 m^2^/g, 340 m^2^/g, 760 m^2^/g, and 1080 m^2^/g for RIAA, W1, W2, and W3 carbons, respectively. The difference in the respective surface area values between the SAXS data and the low-temperature nitrogen sorption data may indicate a pore area inaccessible to nitrogen molecules and thus inaccessible to the adsorbate in adsorption applications. These observations are consistent with the literature reports [36,56].

Figure 2c shows the experimental SAXS curves in the double logarithmic coordinates. The shape of all experimental curves is very similar. As can be seen from the log-log curves the slope of the linear sections of SAXS curves (q_1_ and q_2_ points suggest the Porod linearity) remains in the range of 2  <  α  <  3. This result indicates the fractal distribution of observed heterogeneities. The exact values are α = 3.03, 2.65, 2.67, and 2.62 for RIAA, W1, W2, and W3 carbons, respectively. This may suggest the presence of small-scale volumetric fractal structures created by carbon nanoclusters. The size of the fractal structure can be calculated by the formula *L*  ≈  2π/q2 and equals *L*~4 nm for RIAA and *L*~3.2 nm for other carbons.

**Figure 3:**
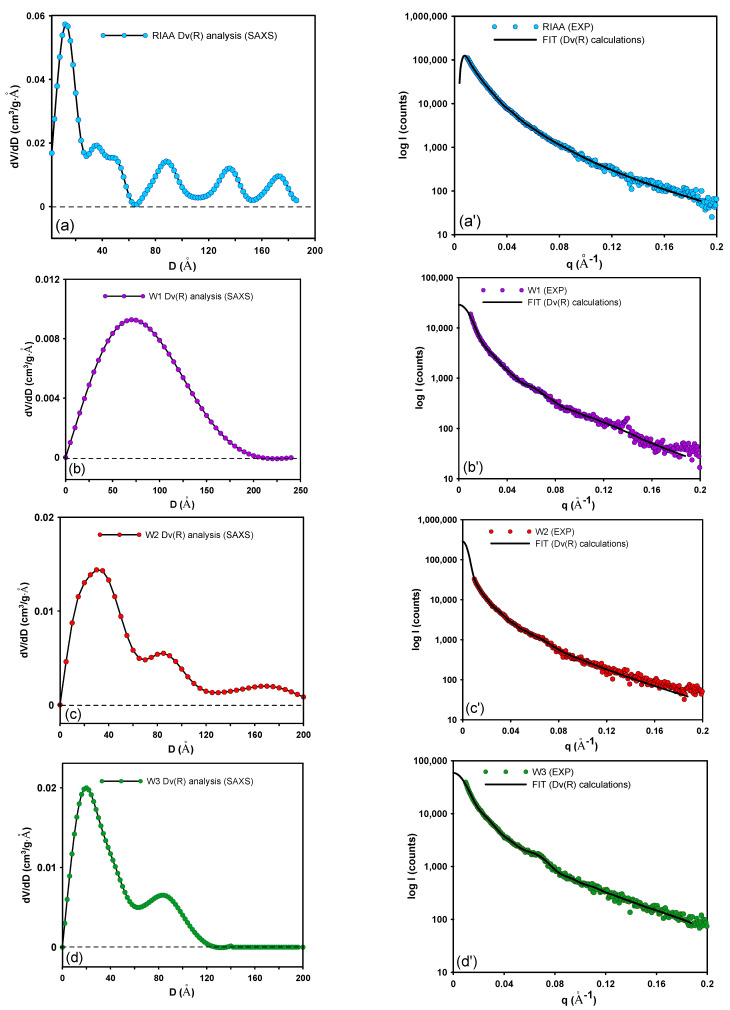
Pore size distribution by the volume analysis Dv(R) for the investigated systems (**a**–**d**). The insets of plots correspond to the fit curves extrapolated for the experimental data (**a’**–**d’**).

Figure 3 shows the volume-weighed particle size distribution Dv(R) from the scattering curve of an ensemble of spherical scattering objects having a homogeneous inner electron density distribution for the investigated systems. In this case, the interparticle interaction effects are not included. The distribution curves indicate that the size of the scatterers is smaller than the resolution limit of the experiment (~50 nm). The Dv(R) function for the microporous carbon RIAA suggests the most frequent radius of particles as 1.36 nm and corresponds to the expected values. For W1, W2, and W3 carbons the obtained results are: 6.3 nm 3.8 nm and 3.3 nm, respectively, suggesting their mesoporous size. The data will remain consistent with the sorption results and may indicate the sorption potential depending on the size of the molecule to be adsorbed.

Additionally, the fit curves were determined during the Dv(R) analysis. The fit curves are the scattering curves (in this case for non-smeared) that correspond to the determined Dv(R) function. In proper solutions, the fit curves should be matched to the parent object (for which the Dv(R) function was determined). In all cases, the fit curves coincide with the experimental SAXS curves and indicate the correctness of the Dv(R) analysis.

**Figure 4:**
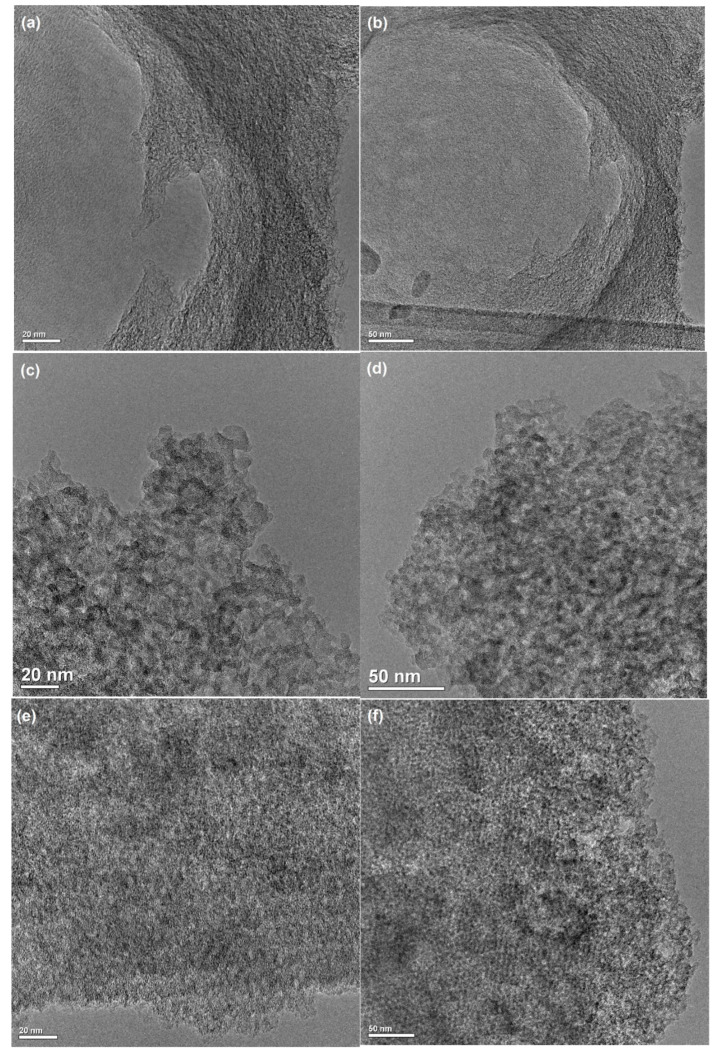

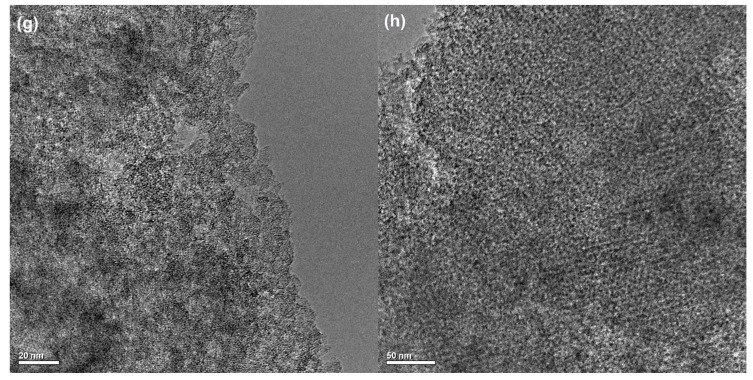
TEM images for the RIAA (**a**,**b**), W1 (**c**,**d**) W2 (**e**,**f**) and W3 (**g**,**h**) carbons.

In Figure 4a–h, the TEM micrographs for the studied carbons are presented. Distinctive differentiation in the structure of the carbon samples is noticeable. One can observe the disordered and curled single carbon layers for the sample RIAA and the structures consisting of almost perfect carbon layers for the W1–W3 samples (typical of well-developed porous material).

**Figure 5:**
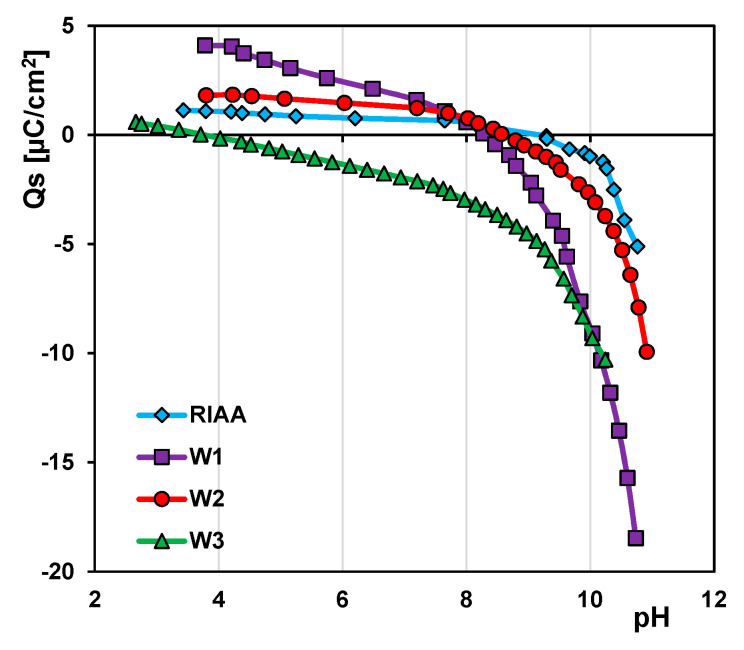
Dependences of surface charge density on pH for the carbonaceous materials.

The dependences of surface charge density vs. pH (Figure 5) show that most of the carbons are basic in nature, with a point of zero charge (pH_PZC_) in the range of 8.3–9.2. While the W3 carbon is acidic, pH_PZC_ is 3.8. This may be explained by some silica residues in the small pores of W3 carbon. In the literature it can be found that pH_PZC_ for silica is in the range of 2.5–3.5 [57]. Generally, under the experimental conditions (neutral pH) all carbons, except W3, exhibit a positive charge.

### 2.2. Adsorption Equilibria

**Figure 6:**
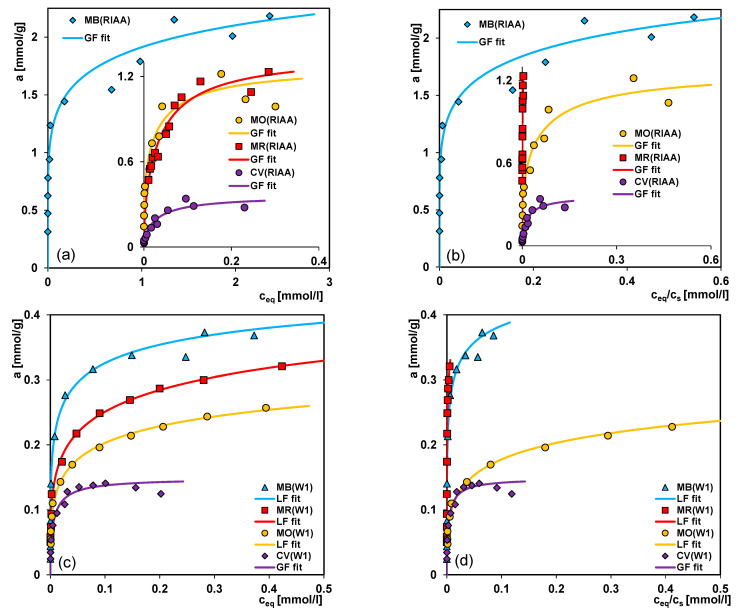

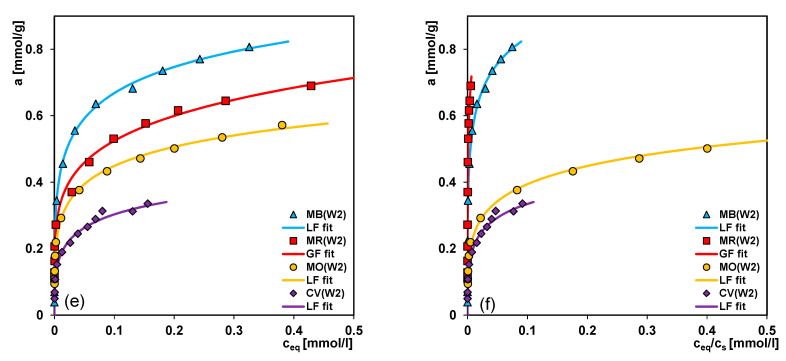
Comparison of the adsorption isotherms for dyes from various structure classes on the activated carbon RIAA (**a**,**b**); W1 (**c**,**d**) and W2 (**e**,**f**) in the standard and reduced coordinate systems.

**Figure 7:**
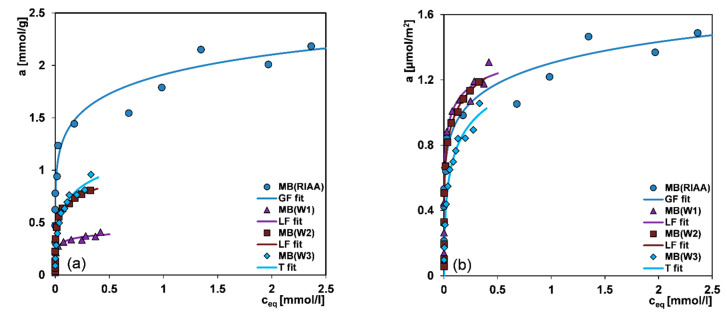

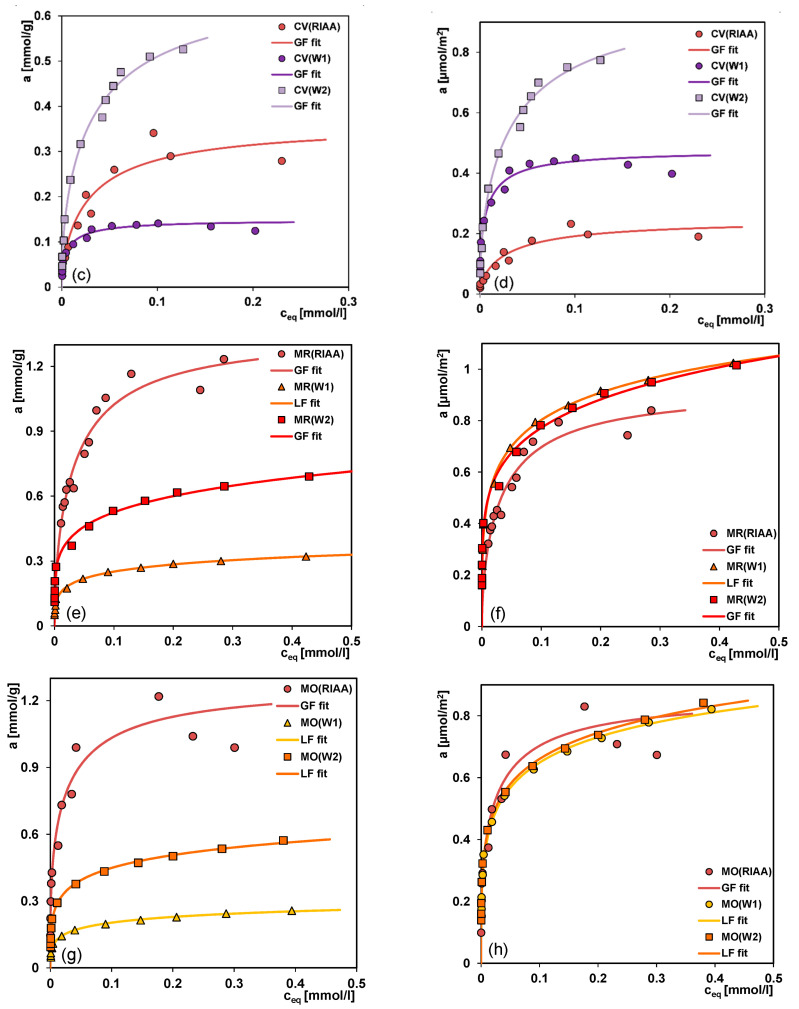
Comparison of adsorption isotherms for methylene blue (**a**,**b**); crystal violet (**c**,**d**); methyl red (**e**,**f**); methyl orange (**g**,**h**) on the studied activated carbons in the standard and reduced coordinate systems.

In the adsorption studies the dyes selected from three structure classes (thiazine, diazene, triarylmethane) of acidic or basic character were used as adsorbates. All dyes were adsorbed on the commercial carbon RIAA, and the mesoporous carbons W1, W2 with similar acid-base properties and differentiated pore structure. While in the case of the W3 carbon with divergent structural and surface properties only methylene blue was adsorbed. The experimental data are drawn in the standard and reduced coordinate system: *a* vs. *c_eq_* and *a* vs. *c_eq_/c_s_*, and in the standard *a* vs. *c_eq_* and reduced *a/S_BET_* vs. *c_eq_* forms (Figure 6a–f, Figure 7a–h, respectively). In Table S1 in Supplementary Materials the most relevant physicochemical properties of dyes such as: ionization constant pK_a_, solubility and partition coefficient P_ow_ are listed. Regarding the effect of the adsorbate (Table S1) and the adsorbent properties (Table 1) on the adsorption process some correlations can be found.

In Figure 6 the adsorption isotherms are compared for 3 adsorbents (RIAA, W1, W2) and 4 dyes (MB, CV, MR, MO). Generally, the important factor in effectiveness of the adsorption process from aqueous solutions is hydrophobicity of the adsorbed compound which can be expressed by the solubility. However, in the case of the studied systems, the course of isotherms reduced by the solubility parameter indicate that the solubility/hydrophobicity is not the main factor determining the adsorption process. Generally, one can find a distinct tendency in the dye affinity for all studied carbons which is as follows: MB > MR >~ MO> CV (thiazine, anionic diazene, triarylmethane derivatives). The weakest adsorption of crystal violet can be explained by the developed molecular structure and size of this dye. Thus, the sieving effect is visible, such a large molecule is excluded from the smallest pores, whereas in the case of other studied dyes their molecules are linear which makes a diffusion into the internal pore structure easier. Taking into account similar surface charge densities for RIAA, W1 and W2 the differences in MB, MO and MR adsorption on a given carbon are evidently associated with their acid-base properties and molecular form. The greater uptake of MB than MO by mesoporous carbonaceous materials was also reported by the authors in the paper [58]. 

In Figure 7a–h the adsorption isotherms for the selected dyes on the microporous carbon RIAA (*S_BET_* = 1468 m^2^/g) and the mesoporous carbons W1, W2 and W3 (*S_BET_* = 313 m^2^/g, 679 m^2^/g, and 908 m^2^/g, respectively) in the standard *a* vs. *c_eq_* and reduced *a/S_BET_* vs. *c_eq_* forms are presented. The second dependence allows to evaluate an adsorption density in a given dye-carbon system. Generally, one can find a distinct correlation between the adsorption capacity and the adsorbent specific surface area and pore size as well as the adsorbate shape and size. In the case of methylene blue (MB), methyl orange (MO) and methyl red (MR) a decrease of adsorption uptake is strictly connected with the carbon specific surface area, and it can be presented as follows: RIAA > W3 (the experimental data only for MB) > W2 > W1. This sequence is consistent with the values of surface area and pore volume of adsorbents. Moreover, analysing the normalized isotherms expressing values of adsorption per surface area unit, one can see that for all carbons and dyes except for CV the curves practically overlap so adsorption is strictly correlated with the specific surface area of adsorbents. For crystal violet (CV) the adsorption changes as follows: W2 > RIAA > W1, and this is a result of developed spatial structure of CV molecule. A deviation of normalized isotherm for the crystal violet/RIAA system is a result of significant contribution of micropores inaccessible to large adsorbate molecules.

Due to aromatic character of all substances some possible adsorption mechanisms can be considered. In the case of the neutral solute form adsorbed on carbon, the dispersion interactions between the π-electrons of the aromatic rings of a solute and the graphene layers of carbon surface predominate. For the ionic form of solutes, adsorption is partly a result of solute–adsorbent electrostatic interactions depending on the dissociation degree. Additionally, in the case of some dyes the electron donor-acceptor interactions or the charge-transfer mechanism should be taken into account.

**Figure 8:**
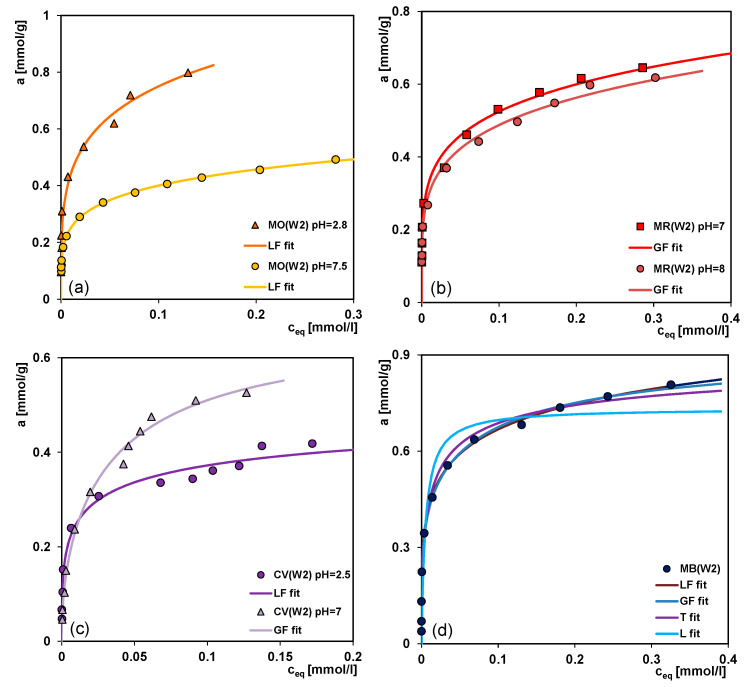
Effect of initial solution pH on methyl orange (**a**); methyl red (**b**) and crystal violet (**c**) adsorption on the W2 carbon. Comparison of isotherms of methylene blue on the W2 carbon fitted by various variants of the Generalized Langmuir (GL) equation (**d**).

Regarding different cationic and anionic solutes (molecular or ionic forms) and the surface charge of adsorbents (all carbons, except W3, exhibit positive charge) it will be necessary to determine the influence of solution pH on the adsorption process. The solution pH plays an important role in the whole adsorption process by affecting the surface charge of the adsorbent and the degree of the ionization of different substances. In addition to the previous experiments conducted at neutral pH, the adsorption process was also studied under the acid and basic conditions. Figure 8a–c shows the comparison of anionic (methyl orange, methyl red) or cationic (crystal violet) dyes adsorption on the W2 carbon at different solution pHs. Generally, for the anionic compounds, adsorption increases with the decreasing pH while for the cationic compound, the situation is reverse, adsorption increases as pH increases. Methyl orange is strongly adsorbed in the acidic solution of pH = 2.8. Under these conditions, the adsorbate is present only in the form of undissociated molecules which show significant affinity for the surface of carbon adsorbent of hydrophobic character. Much lower adsorption at pH = 7.5 is observed when anions interact weakly with the activated carbon. The differentiation in adsorption of methyl red at pHs of 7 and 8 is a result of difference in charge of the adsorbent surface. The higher pH the lower the charge of carbon surface occurs so the electrostatic interactions get weaker.

Lower adsorption of crystal violet at pH = 2.5 is probably due to the presence of H^+^ ions competing with the cation groups of the dye for adsorption sites. Moreover, in the acidic solution the electrostatic repulsion occurs between the positive crystal violet ions and the positively charged surface of activated carbon (PZC = 8.4). As the surface charge density decreases with the increasing pH of solution, the electrostatic repulsion becomes smaller, resulting in the adsorption increase. Moreover, according to the literature [59] at higher solution pH, the association of dye cations on the solid takes place easily. This can favour adsorption.

For the analysis of the experimental adsorption, the Generalized Langmuir (GL) isotherm (Table S4) was used which for the specific values of the heterogeneity parameters is reduced to four simpler equations: Langmuir (L, *m* = *n* = 1), Langmuir-Freundlich (LF, 0 < *m* = *n* ≤ 1), Generalized Freundlich (GF, 0 < *m* ≤ 1, *n* = 1) and Tóth (T, *m* = 1, 0 < *n* ≤ 1). These isotherms were obtained from the global integral equation, assuming the Langmuir isotherm as a local one. The GL equation corresponds to the quasi-Gaussian distribution function of the adsorption energy with the heterogeneity parameters *m* and *n*, characterizing broadening of this function to higher (*m* < 1) and lower (*n* < 1) adsorption energies (when *m* ≠ *n*, the energy distribution is asymmetrical, the smaller *m* and *n*, the greater heterogeneity). The Tóth equation corresponds to the quasi-Gaussian distribution function of the adsorption energy expanded in the direction of low energy while the isotherm GF relates to the exponential function with a characteristic minimum of energy. The applied equations show a slightly different behaviour in the range of low and high adsorption. Only the Tóth isotherm is reduced at low concentrations, $\underset{c\to 0}{\lim}\theta \;=0$ to the Henry’s equation, $\theta =Kc_{eq}$ whereas the other isotherms to the classical Freundlich equations, $\theta ={\left(Kc_{eq}\right)}^{m}$. At high concentrations, $\underset{c\to \infty }{\lim}\;\theta =1$, all equations show the typical Langmuir behaviour, however, the equation GF achieves the maximum adsorption very quickly. The exemplary comparison of the adsorption isotherms of the Generalized Langmuir equation and the values of their parameters for the MB/W2 system are presented in Figure 8d and Table 3 (pink background), respectively.

On the basis of the values of 1−*R^2^* and *SD(a)*, the best fitting equation for all studied systems was chosen and the respective data are presented in Table 3. A good fit using the GF, LF or Tóth equations was obtained. The values of heterogeneity parameters in most cases are much lower than unity, reflecting the significant impact of energetic heterogeneity. Large differentiation of values of equilibrium constants indicates various adsorption affinities of the dyes for the carbon surface. In all cases good agreement between the experimental points and fitted lines is observed.

Some studies on adsorbing performance of various materials for the studied dyes and the corresponding maximum adsorption capacities have been reported [25,26,33,36,60,61,62,63,64,65,66], which are listed in Table 4. Compared with referred materials, the mesoporous carbons obtained by impregnation of well-organized silicas proved to be similar or even more efficient adsorbents. Only for graphene oxide intercalated montmorillonite nanocomposite [26] and natural organic matter rich clays adsorption effectiveness is much higher [65].

### 2.3. Adsorption Kinetics

**Figure 9:**
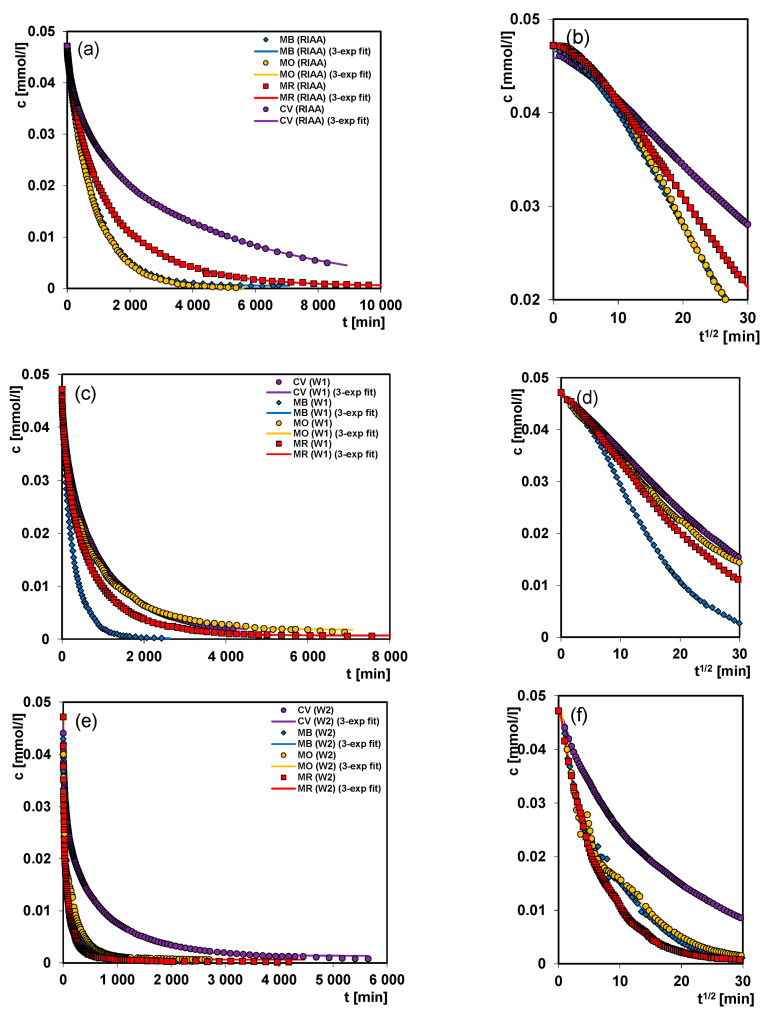
Comparison of the adsorption kinetics for various dyes on the carbonaceous materials RIAA (**a**,**b**); W1 (**c**,**d**); W2 (**e**,**f**) at the coordinates: relative concentration ~ time and relative concentration ~ square root of time. The lines correspond to the fitted multi-exponential equation.

In order to study the influence of dye and carbon properties on the adsorption rate, the kinetic curves were measured by cyclic collecting the scans where each spectrum corresponded to a single experimental point. In Figure S1 in Supplementary Materials the exemplary spectra obtained during the adsorption of methylene blue on the W3 carbon are presented. In Figure 9a–f the experimental data in the two coordinate systems: concentration (*c*) ~ time (*t*) and concentration (*c*) ~ root of time (*t*^1/2^) are shown.

From the analysis of the concentration profiles obtained for the adsorption of various dyes on the RIAA carbon, one can state that the rates of the process are arranged in the following series from the fastest: MB ~ MO > MR > CV. Generally, the adsorption rate depends largely on a molecular size of solutes. MR shows a certain deviation from this rule which can be explained by its high solubility which largely decreases affinity for the hydrophobic surface of adsorbent. In the case of dye with the largest particle size, CV, one can consider two effects: the hindered diffusion of adsorbate in larger pores and exclusion from small pores like for the RIAA carbon. Thus, both the rate and decolorization efficiency for this system are the smallest.

The analysis of the kinetic curves for dyes adsorption on the mesoporous carbons W1 and W2 allows to confirm that a molecular size of adsorbate is one of the main factors determining a rate of adsorption process. In the case of W1 carbon having pores with a larger size this effect is insignificant in relation to smaller adsorbates, like MB for which the adsorption rate is greater than for the smaller MR as a result of different physicochemical properties. In the case of mesoporous carbon W2 with the pores of smaller size, one can see quite clearly the effect of hindered diffusion of large adsorbates like CV which results in the rate decrease.

**Figure 10:**
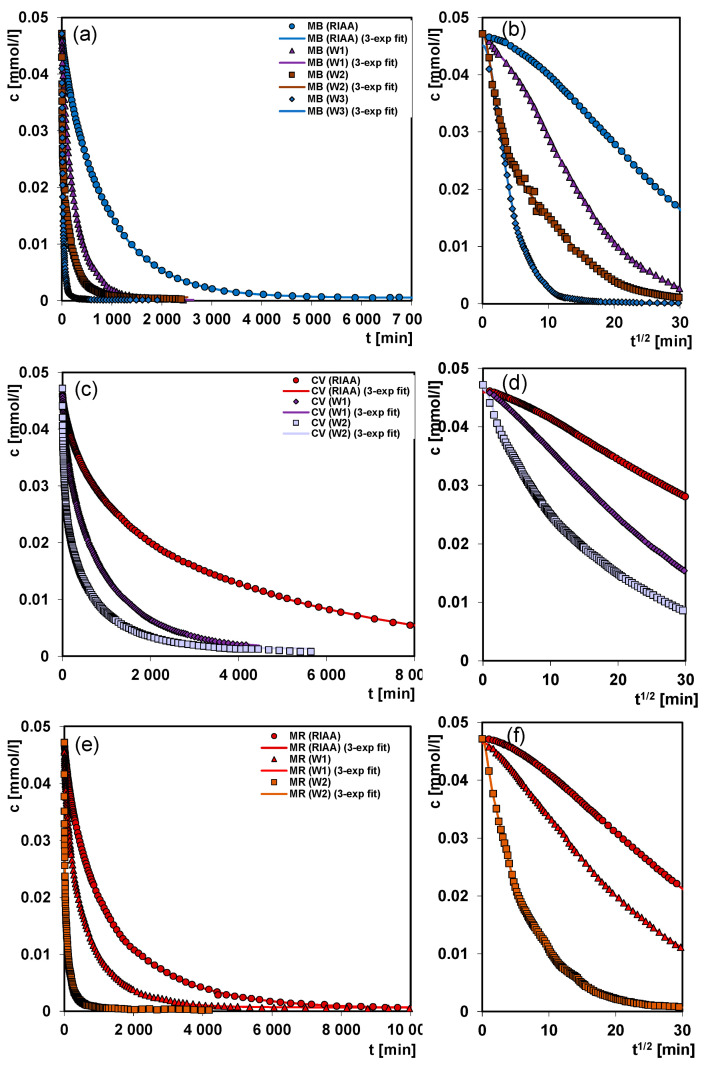

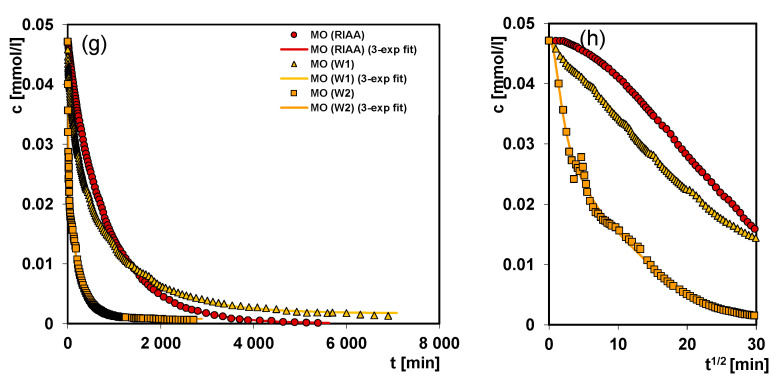
Comparison of adsorption kinetics of methylene blue (**a**,**b**); crystal violet (**c**,**d**); methyl red (**e,f**) and methyl orange (**g**,**h**) on the activated carbons at the coordinates: relative concentration ~ time and relative concentration ~ square root of time.

In Figure 10a–h the kinetic dependences for the adsorption of the selected dye (methylene blue, crystal violet, methyl red and methyl orange) on the mesoporous carbons W1, W2, W3 and the microporous carbon RIAA are plotted. Considering the course of the adsorption processes on the mesoporous carbons there can be seen that the fastest adsorption occurs on the mesoporous carbons with a larger specific surface area. The slowest adsorption process on the microporous carbon RIAA is observed, despite the largest surface area. The critical factor of slow adsorption on this adsorbent is its microporous structure which disturbs the diffusion of large dye molecules. This is particularly visible in the system with crystal violet as an adsorbate of large size molecules, where time of the experiment is insufficient to achieve the adsorption equilibrium. Generally, the rate of dyes adsorption from the aqueous solutions on carbons decreases in the order of W3 > W2 > W1 > RIAA. Mesoporous carbon W3 has both the largest surface area and total pore volume. The rapid course of the dyes’ adsorption using the adsorbents W2 and W3 is confirmed by a nearly straight dependency of concentration versus time for a predominant part of the process.

The experimental kinetic profiles for all adsorption systems were analysed using the kinetic models such as: the first order equation (FOE), the second order equation (SOE), the mixed 1.2-order equation (MOE) and the multi-exponential equation (m-exp). In Table S2 in Supplementary Materials the comparison of the parameters calculated according to the equations for all experimental systems is given. A very good correlation between the experimental data and the multi-exponential equation was obtained that is confirmed by the *SD*(*c*/*c*_0_) and 1−*R*^2^ values.

**Figure 11:**
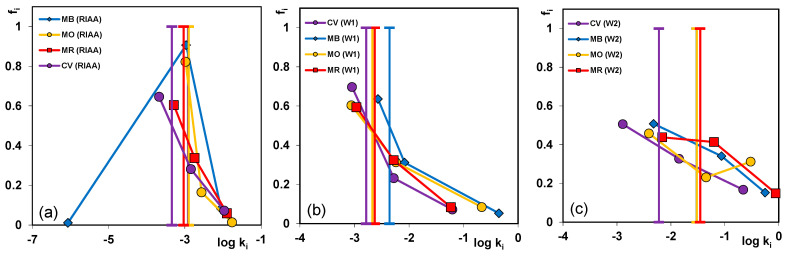
Parameter spectra for the multi-exponential equation for the dye adsorption kinetics on the activated carbon RIAA (**a**); W1(**b**) and W2 (**c**).

For each experimental system the adsorption half-times *t*_0.5_ were determined, i.e., the time required to adsorb half of *a_eq_*. The parameter for various terms of multi-exponential equation was obtained from the relationship: $t_{0.5}=\left(ln\;2\right)/k_{i}$ while for the total kinetics it was determined numerically (Table S2 in Supplementary Materials). In Figure 11a–c the parameters as the spectrum *f_i_* vs. *log k*_*i*_ were plotted. Each spectrum reflects shares of terms characterized by various rate coefficients.

Most significant differentiations in respect to both the shape and calculated values of the rate coefficients for the system with the RIAA carbon were obtained. Distributions of kinetic constants are broad that indicates that the dyes adsorption process proceeds with a changing rate in the time range of the experiment. Faster kinetics on the mesoporous activated carbon W2 corresponds to the higher share of kinetic constants with higher magnitudes, compared to the RIAA and W1 carbons (high share *f_i_* for low *k_i_*).

**Figure 12:**
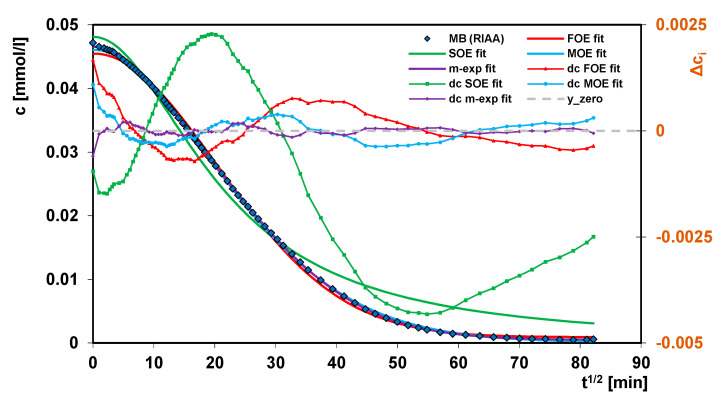
Comparison of the adsorption kinetics for methylene blue on the activated carbon RIAA fitted to the FOE, SOE, MOE and m-exp. equations.

In Figure 12 the exemplary experimental data (MB/RIAA), fitted with the FOE, SOE, MOE and m-exp kinetic equations as well as their deviations in the Weber-Morris linear coordinates are plotted. It can be seen that the best fitting for the multi-exponential equation was obtained, its SD is about 6 and 27 times better than for the FOE and SOE equations, respectively. The fitting parameters for the MOE equation are three times worse than for multi-exp. For all equations with the exception for SOE the greatest deviations in the initial step of the kinetic curves are observed. Comparing the fitting quality for all studied systems, the multi-exponential equation works satisfactorily, but of the other equations it is impossible to indicate the best or the worst one, which proves diversity of the adsorption systems.

### 2.4. Thermal Degradation of the Systems

The results are presented and discussed in Supplementary Materials.

## 3. Methods and Calculation Procedures

### 3.1. Mesoporous Carbon Preparation

The synthesis of mesoporous carbons was modification of the hard-templating method [67], i.e., impregnation of mesoporous silicas with the carbon precursor followed by the carbonization process. In the synthesis of mesoporous silicas, the amphiphilic triblock copolymers: Pluronic PE9400 and Pluronic PE10500 (BASF Corporation, Poland) were used. The essential information about Pluronics is presented in Table S3 in Supplementary Information. The process was conducted under the acidic conditions (1.6M HCl), where the copolymers, tetraethylorthosilicate (TEOS) and 1.3.5-trimethylbenzene (TMB) play a role of pore-creating agents, a silica source, and a pore expanding agent, respectively. The composition of each reacting mixture was fixed: the polymer concentration—2.7%, the mass ratio of polymer to 1.3.5-trimethylbenzene—1:1, the mass ratio of polymer to tetraethylorthosilicate—1:3.4. First, the reacting mixtures were aged in the autoclaves (the temperatures and times of ageing process are given in Table S4 in Supplementary Materials). The obtained silicas were washed with distilled water, dried and calcined. The next step of the synthesis comprised a two-stage impregnation of silicas with the aqueous solution of carbon precursor containing H_2_SO_4_ as a catalyst (the mass ratio of silica to sucrose in the first and second stages was 1:2.6 and 1:1.53, respectively). The obtained intermediate products were carbonized in the nitrogen atmosphere, followed by removal of the silica skeleton with the KOH solution. The parameters of mesoporous carbon synthesis are collected in Table S4 in Supplementary Materials.

### 3.2. Adsorbates

In the studies, the cationic dyes: (methylene blue, MB; crystal violet, CV), and the anionic dyes (methyl orange, MO; methyl red, MR), belonging to the derivatives of: thiazine (MB), diazene (MO, MR), triarylmethane (CV), were used. They were purchased from the Sigma-Aldrich or POCH (Poland). The physicochemical properties and structure of the dyes are presented in Table S1 and Figure S4, respectively in Supplementary Materials.

### 3.3. Material Characterization

#### 3.3.1. Nitrogen Adsorption/Desorption Measurements

The structure characteristics of the studied carbons were obtained from the low-temperature adsorption/desorption of nitrogen (ASAP 2020, Micromeritics). On the basis of the measured nitrogen adsorption/desorption isotherms there were calculated the values of the following parameters: the BET specific surface area (*S_BET_*), the total pore and micropore volumes (*V_t_* and *V_mic_*), the external surface area (*S_ext_*). The calculations of the pore size distributions (PSD) followed the Barrett, Joyner and Halenda (BJH) procedure. The pore diameters from the PSD maximum (mode, *D_mo_*) were estimated. The mean hydraulic pore diameters were calculated from the BET surface areas and pore volumes, *D_h_ = 4V/S*. The Horvath-Kawazoe method was applied for the micropore structure analysis.

#### 3.3.2. Small-Angle X-ray Scattering (SAXS)

The SAXS measurements were performed using an Empyrean (PANalytical) diffractometer. The capillary mode as reflection geometry was applied as SAXS configuration for single scan procedures. All measurements were performed in the 2θ range of 0.13–4 degrees. The generator settings of 40 kV and 40 mA were applied during the measurements. The incident beam path was composed of a line focus type, W/Si, graded X-ray mirror with the elliptic shape. The primary beam was measured using a beam attenuator Cu 0.2 mm. The PIXcel1D detector, and the receiving slit with the 0.05 mm active length were used during the measurements. The length of the scattering vector *q* is defined as *q* = 4πsinθ/λ, where 2θ is the scattering angle, λ is the X-ray wavelength (1.5418 Å). The air scattering measure with an empty sample holder for the background correction was performed. The measurement times were 2300 s for each sample. The Dv(R) calculations as the indirect Fourier transformation technique applied in the EasySAXS software (PANalatycal) were used. In this case, the applied algorithm based on the Tikhonov’s regularization method was accomplished.

#### 3.3.3. Potentiometric Titration Measurements

In order to evaluate the acid-base nature of carbons a potentiometric titration measurement of suspension in the NaCl electrolyte with the ionic strength of I = 0.1 mol/L was used [68]. Based on the experimental relations pH = f(V_titr_), the surface charge of the solids was determined using the equation:

(1) $$q_{s}=\frac{F\;\Delta n_{H^{+}}}{S_{BET}}$$

where: *q*_*s*_ is the surface charge,$\;\Delta n_{H^{+}}$ is the number of *H*^+^ moles per 1 g of material, *F* is Faraday’s constant, *S_BET_* is the specific surface area.

#### 3.3.4. Transmission Electron Microscopy (TEM)

The carbon surface topography was analysed by the transmission electron microscopy (Titan G2 60–300 kV FEI Company). The pretreatment stage comprised: grinding samples into a fine powder, pouring with ethanol and ultrasound homogenization.

#### 3.3.5. Thermal Analysis

Thermal analysis was carried out on a STA 449 Jupiter F1 apparatus, (Netzsch, Selb, Germany) under the following operational conditions: heating rate of 10 °C/min, dynamic atmosphere of synthetic air (50 mL/min), temperature range of 30–950 °C, sample mass ~15 mg, sensor thermocouple type *S* TG-DSC. The gaseous products emitted during the decomposition of samples were analyzed by the FTIR (FTIR spectrometer, Bruker, Ettlingen, Germany) technique.

### 3.4. Adsorption Experiment

#### 3.4.1. Adsorption Equilibrium

Adsorption isotherms were measured using a static method. In the case of mesoporous carbons (W1, W2 and W3), the known amount of adsorbent was contacted with a small volume of water or electrolyte with the fixed pH and degassed under vacuum. Then the dye solution of defined concentration was added and the sample was placed in a thermostatic shaker. After attaining the adsorption equilibrium, the solute concentration was calculated using the spectrophotometric absorption data (Cary 4000, Varian Inc., Australia) measured at λ_max_(MB) = 664 nm, λ_max_(CV) = 582 nm, λ_max_(MO) = 464 nm, λ_max_(MR) = 429 nm, respectively. In the next stage, a certain portion of concentrated adsorbate was added to a sample and after attaining the adsorption equilibrium the dye concentration was measured. The procedure was repeated several times in order to measure the adsorption isotherms over a wide range of concentrations.

For the commercial activated carbon RIAA, the experiment was slightly altered. First, the initial dye solutions in a wide range of concentrations were prepared from the stock solution of adsorbate. Next, to the carbon samples after degassing under vacuum, the dye solutions were added and placed in a thermostated shaker. After attaining the adsorption equilibrium, spectrophotometric measurements were performed. The adsorbed amounts of substances were calculated from the mass balance equation.

Most adsorption experiments were conducted at neutral pH, however, some additional measurements were made at selected pH values. The pH of the solutions was maintained by adding hydrochloric acid or sodium hydroxide.

The analysis of the equilibrium data on the basis of adsorption model on the energetically heterogeneous solids by applying the Generalized Langmuir isotherm equation (GL) was conducted [69]. The isotherm equation and its simplified forms are collected in Table S5 in Supplementary Materials.

#### 3.4.2. Adsorption Kinetics

The kinetic batch experiments were performed by means of a UV/Vis spectrophotometer (Cary 100, Varian Inc.). The dye aqueous solution of a given initial concentration was contacted with the known amount of adsorbent in a measurement vessel. The solution was stirred during the experiment using a magnetic stirrer, the solution samples were collected to a flow cell at the defined time intervals. The measured concentration vs. time profiles were calculated from the recorded spectra and analysed using the well-known kinetic equations: FOE/PFOE [70,71,72], SOE/PSOE [73], the mixed 1.2-order (MOE) [74], and the multi-exponential (m-exp) equations [75]. The general and/or linear forms of the applied kinetic equations are listed in Table S6 in Supplementary Materials [76].

## 4. Conclusions

The series of mesoporous carbonaceous materials with well-organized structure was synthesized, and their structural, surface, and adsorption properties were investigated, and compared with those of the microporous activated carbon. The obtained materials show differentiated fairly homogeneous porosity: W1 – *S_BET_* = 313 m^2^/g, *V_t_ =* 0.34 cm^3^/g, *D*_*mo*_ = 5.3 nm; W2 – *S_BET_* = 679 m^2^/g, *V_t_ =* 0.71 cm^3^/g, *D*_*mo*_ = 4.2, and W3 – *S_BET_* = 9.08 m^2^/g, *V_t_ =* 0.75 cm^3^/g, *D_mo_* = 3.5 nm. The SAXS technique indicates the pore size for the W1, W2, and W3 samples (6.3 nm, 3.8 nm, and 3.3 nm, respectively) that is in good agreement with the nitrogen sorption results. SAXS analysis confirms some degree of structural order for all mesoporous systems. Basing on the TEM analysis ordered structure consisting of almost perfect carbon layers for the W1 and W3 samples is confirmed. Most of the studied carbons are basic in nature (pH_PZC_ in the range of 8.3–9.2), whereas the W3 carbon is acidic (pH_PZC_ = 3.8).

The adsorption properties of the mesoporous carbons and microporous activated carbon were compared towards selected dyes. A distinct tendency in the dye affinity for all studied carbons is found: MB (methylene blue) > MR (methyl red) >~ MO (methyl orange) > CV (crystal violet). The weakest adsorption of crystal violet is correlated with its developed molecular structure and size. The differences in MB, MO and MR adsorption on a given carbon are associated with their acid-base properties. For MB, MO and MR adsorption on different carbons a decrease of adsorption uptake is connected with the carbon specific surface area: RIAA > W3 (the experimental data only for MB) > W2 > W1. For crystal violet the adsorption effectiveness is different: W2 > RIAA > W1, which is well correlated with developed spatial structure of its molecules.

Basing on the kinetic studies on a given material the rates of adsorption process change from the fastest in the series: MB ~ MO > MR > CV. The slowest adsorption of crystal violet one can explain by the hindered diffusion of adsorbate in the carbon pores. In comparison to the mesoporous materials for the microporous carbon RIAA the slowest adsorption process is observed for all adsorbates which is connected with difficult diffusion of large dye molecules into the small micropores. The adsorption rates decrease in the order of W3 > W2 > W1 > RIAA. The multi-exponential equation gives a very good correlation with the experimental data. The adsorption half-times determined for methylene blue equal: 13 (W3) > 23 (W2) > 158 (W1) > 572 min (RIAA). Whereas, the values of the discussed parameter for methyl red/W2/W1/RIAA; methyl orange /W2/W1/RIAA and crystal violet /W2/W1/RIAA systems are as follows: 20 > 294 > 745 min.; 23 > 362 > 549 min.; 115 > 423 > 1516 min. The observed relatively slow kinetics for the W1 carbon despite its greatest share of the mesopores in the total porosity proves that the process is very complex and is also dependent on other adsorbent properties e.g., particle size, pores shape, surface chemistry, hydration degree.

The spectrum of m-exp parameters for the system with the RIAA carbon reveals most significant differentiations in respect to both the shape and calculated values of the rate coefficients. Generally, the broad distributions of kinetic constants indicate that the dye adsorption process is differentiated in time and proceeds with a changing rate during the experiment. Faster kinetics on the mesoporous activated carbon W2 corresponds to the higher share of kinetic constants with higher magnitudes.

The thermal analysis for the carbonaceous material loaded with methylene blue in comparison to pure adsorbent reveals a shift of the experimental curves towards lower temperatures. (The results are presented in Supplementary Materials). Analysis of volatile products released during the thermal degradation allows to determine the interactions in adsorption system by attributing the characteristic bands to a specific bonding. The signals recorded for SO_2_ and HCN confirm the adsorption of the dye molecules on carbon surface.

Taking into account that kinetics of adsorption process may be a critical and decisive factor determining usefulness of the technique on a large scale in technological applications, all these studies clearly indicate that the obtained mesoporous carbonaceous materials are efficient adsorbents for the removal of various dyes from industrial wastewaters. Good results of the equilibrium and kinetic measurements show that the W3 carbon is a particularly promising among others.

## Figures and Tables

**Table 1:** The values of parameters characterizing the porous structure of adsorbents.

Carbon	*S_BET_* [m^2^/g]	*V*_*t*_ [cm^3^/g]	*V*_*mic*_ (t-plot) [cm^3^/g]	Dv(R) [nm] SAXS	*D*_*h*_ [nm]	*D*_*mo*_ (des. BJH) [nm]
RIAA	1468	0.80	0.31	1.36	2.2	1.8
W1	313	0.34	0.06	6.3	4.3	5.3
W2	679	0.71	0.08	3.8	4.2	4.2
W3	908	0.75	0.03	3.3	3.3	3.5

**Table 2:** SAXS structural parameters for the investigated carbon samples.

Sample	R_Dv(R)_^a^[Å]	Porod Approximation				Specific Surfaces Area	
		*K* ^b^	Q ^c^[Å^−1^]	C_0_^d^	S/V[Å^−1^]	S_BETADS/DES_[m^2^/g]	*S_SAXS_*^e^[m^2^/g]
RIAA	13.6	0.53	−7.02	38.31	0.078	1468	1568
W1	63.0	0.16	7.53	11.78	0.017	313	340
W2	38.5	0.25	13.3	23.70	0.038	679	760
W3	33.0	0.40	13.30	23.72	0.054	908	1080

^a^ The volume-weighed object size distribution Dv(R) as the maximum value of the function. ^b^ Porod constant is proportional to the surface area and the square of the electron density contrast. ^c^ Scattering invariant *Q* is proportional to the mean-square density fluctuation of scattering volume. *Q* = 2π²·Δ*ρ*²·V where V is the volume and Δ*ρ* is the scattering contrast. For calculation of *Q* invariant the scattering intensities should be extrapolated to *q* = 0 and also towards large *q*. ^d^ Background constant which illustrates the asymptotic decay of the SAXS curve at the high *q* values. ^e^ Surface area by SAXS calculated from the equation $S_{SAXS}=\frac{10000\cdot \frac{S}{V\;}\left[{Å}^{-1}\right]}{d\;\left[\frac{g}{cm^{3}}\right]}$ where S/V—the surface to volume ratio calculated from the distribution curve, *d*—the mass density of material.

**Table 3:** The parameters of the Generalized Langmuir equation for the studied systems.

System	Isotherm	*a*	*m*	*n*	log *K*	1−*R*^2^	*SD*(*a*)
MB(RIAA)	GF	2.79	0.16	1	−1.01	0.024	0.119
MB(W1)	LF	0.53	0.33	0.33	1.60	0.012	0.017
MB(W2)	LF	1.56	0.29	0.29	0.58	0.003	0.018
MB(W2)	GF	0.92	0.23	1.00	0.57	0.004	0.021
MB(W2)	T	0.96	1.00	0.42	3.00	0.032	0.056
MB(W2)	L	0.73	1.00	1.00	2.28	0.082	0.085
MB(W3)	T	1.64	1	0.37	2.14	0.013	0.034
MO(RIAA)	GF	1.28	0.31	1	1	0.040	0.080
MO(W1)	LF	0.43	0.35	0.35	0.86	0.007	0.007
MO(W2)	GF	0.7018	0.23	1	0.23	0.005	0.014
MO(W2) pH = 2.8	GF	1.29	0.25	1	0.11	0.061	0.071
MO(W2) pH = 7.5	GF	0.72	0.21	1	−0.17	0.002	0.007
MR(RIAA)	GF	1.37	0.53	1	1.14	0.052	0.064
MR(W1)	GF	0.38	0.24	1	0.35	0.059	0.027
MR(W2)	T	0.84	1	0.38	3	0.109	0.071
MR(W2) pH = 8	GF	0.809	0.21	1	0.06	0.0919	0.065
CV(RIAA)	GF	0.36	0.56	1	1.24	0.073	0.033
CV(W1)	GF	0.15	0.37	1	1.64	0.018	0.007
CV(W2) pH = 2.5	LF	0.57	0.39	0.39	1.71	0.017	0.019
CV(W2)	GF	0.67	0.47	1	1.09	0.007	0.016

**Table 4:** Comparison of the maximum adsorption capacity of different adsorbents towards the studied dyes.

Adsorbent	Dye	Maximum Sorption Capacity	Ref.
activated biochar/ rice straw precursor	methylene blue	0.28 mmol/g (91 mg/g)	[60]
lignin/chitosan composite	methylene blue	0.11 mmol/g (36 mg/g)	[33]
raphiafiber	methylene blue	0.11 mmol/g (35 mg/g)	[61]
activated carbon/ mesoporous silica template	methylene blue	0.53–1.64 mmol/g	present study
aminated pumpkin seed	methyl orange	0.61 mmol/g (200 mg/g)	[62]
amino functionalized Zr-based MOFs	methyl orange	0.45 mmol/g (148 mg/g)	[63]
h-MoS_2_ microspheres	methyl orange	0.12 mmol/g (38 mg/g)	[51]
activated carbon/ mesoporous silica template	methyl orange	0.43–0.70 mmol/g	present study
Ag@Fe nanocomposite	methyl red	0.43 mmol/g (125 mg/g)	[64]
natural organic matter rich clays	methyl red	1.36 mmol/g (397 mg/g)	[65]
activated carbon/apple shell precursor	methyl red	0.78 mmol/g (227 mg/g)	[66]
activated carbon/ mesoporous silica template	methyl red	0.38–0.84 mmol/g	present study
functionalized multiwalled carbon nanotubes	crystal violet	0.75–0.88 mmol/g	[25]
activated carbon/ pistachio shells precursor	crystal violet	0.12 mmol/g	[36]
graphene oxide intercalated montmorillonite nanocomposite	crystal violet	1.83 mmol/g (746mg/g)	[26]
activated carbon/ mesoporous silica template	crystal violet	0.15–0.67 mmol/g	present study

## Data Availability

The data are available by corresponding author.

## Supplementary Materials

The following are available online. Figure S1: Exemplary absorption spectra in the Vis range measured during the adsorption process for the MB (W3) system; Figure S2: Comparison of the TG, DTG and DSC curves measured for methylene blue (a), W3 (b) and methylene blue /W3 systems (c); Figure S3: FTIR spectra of gas products of pyrolysis of methylene blue, W3 and methylene blue /W3 system at the temperatures corresponding to the process rate maxima; Figure S4: Chemical structure of the dyes: (a) methylene blue; (b) crystal violet; (c) methyl orange; (d) methyl red; Table S1: Physicochemical properties of the dyes; Table S2: Comparison of the parameters of the FOE, SOE, MOE and multi-exponential kinetic equations for the studied systems; Table S3: Physicochemical properties of the amphiphilic triblock copolymers used in the silica synthesis; Table S4: Parameters of the synthesis of mesoporous silicas and carbons; Table S5: Generalized Langmuir isotherm equation (GL) and its simplified forms; Table S6: Various kinetic equations applied in the study.

## Sample Availability

Samples of the activated carbons are available from the authors.
