# Supplementary material for: Mesoporous Carbons of Well-Organized Structure in the Removal of Dyes from Aqueous Solutions
Source: Molecules. 2021 Apr 9;26(8):2159. doi: 10.3390/molecules26082159 (PMC8069419; doi:10.3390/molecules26082159)
Supplement: Supplementary file 1 [file molecules-26-02159-s001.pdf]

molecules

MDPI

Supplementary material for the paper:

Mesoporous Carbons of Well-Organized Structure in the Removal of Dyes from Aqueous Solutions

Magdalena Blachnio*, Anna Derylo-Marczewska, Szymon Winter, Malgorzata Zienkiewicz-Strzalka

Faculty of Chemistry, Maria Curie-Sklodowska University, M. Curie-Sklodowska Sq. 3, 20-031 Lublin, Poland; annad@hektor.umcs.lublin.pl (A.D.-M.); winter1972@wp.pl (S.W.); malgorzata.zienkiewicz@poczta.umcs.lublin.pl (M.Z.-S.)

* *Correspondence:* magdalena.blachnio@poczta.umcs.lublin.pl (M.B.); Tel.: +48-8153-756-37 (M.B.)

Results and Discussion

Adsorption Equilibria

Table 1. Physicochemical properties of the dyes.

Dye (code)	Chemical formula	Molecular weight [g/mol]	Ionization constant, pK_a	Water solubility [%]	log P_{ow}
Methylene blue (MB)	$C_{16}H_{18}N_3ClS$	319.85	2.6; 11.2[1]	4.36[2]; 4[3]	-0.9[4]
Crystal violet (CV)	$C_{25}H_{30}N_3Cl$	407.99	0.8[5]; 9.4[6]	1.7[7]	0.51[8]
Methyl orange (MO)	$C_{14}H_{14}N_3O_3SNa$	327.34	3.4[1]; 3.7[9]	0.15[10]; 0.5[11]	-0.66[8]
Methyl red (MR)	$C_{15}H_{14}N_3NaO_2$	291.30	2.3; 5[1]	80[12]	0.03[8]

[1.] Sabnis, R.W. *Handbook of Biological Dyes and Stains: Synthesis and Industrial Applications*, 1st ed.; John Wiley & Sons, Inc.: Hoboken, New Jersey, United States, 2010. [2.] Peters, A.T., Freeman, H.S. *Physico-Chemical Principles of Color Chemistry*, 1st ed.; Springer International Publishing: Cham; 1996.[3] Troy, D.B., Beringer, P. *The Science and Practice of Pharmacy*, 21st ed.; Lippincott Williams & Wilkins.: Philadelphia, United States, 2006. [4.] Perotto, G.; Sandri, G.; Pignatelli, C.; Milanesi, G.; Athanassiou, A. Water-based synthesis of keratin micro- and nanoparticles with tunable mucoadhesive properties for drug delivery (Electronic Supporting Information). [5.] Nalewaja, J.D.; Goss, G.R.; Tann, R.S. *Pesticide Formulations and Application Systems*; American Society for Testing and Materials: West Conshohocken, 1997. [6.] Crini, G., Badot P.-M. *Sorption Processes and Pollution: Conventional and Non-conventional Sorbents*, 1st ed.; Besançon Presses universitaires de Franche-Comté, 2010. [7.] Suvarna, K.; Layton, C.; Bancrof J.D. *Bancroft's Theory and Practice of Histological Techniques*, 7th ed.; Churchill Livingstone: London, Great Britain, 2012. [8.] Toxicology Data Network, https://toxnet.nlm.nih.gov/.[9.] Sohn, Y.S. *Photoelectrocatalytic Degradation of Organic Dye Molecules on Titanium*, 1st ed.; Proquest, Umi Dissertation Publishing: Charleston SC, United States, 2012. [10.] Gangolli, S. *The Dictionary of Substances and Their Effects*, 3rd ed.; Royal Society of Chemistry, Great Britain, 2005. [11.] Safety Data Sheet of the product - Methyl orange (C.I. 13025) by Merck KGaA. [12.] Safety Data Sheet of the product - Methyl red sodium salt (C.I. 13020) by Merck KGaA.

Adsorption kinetics

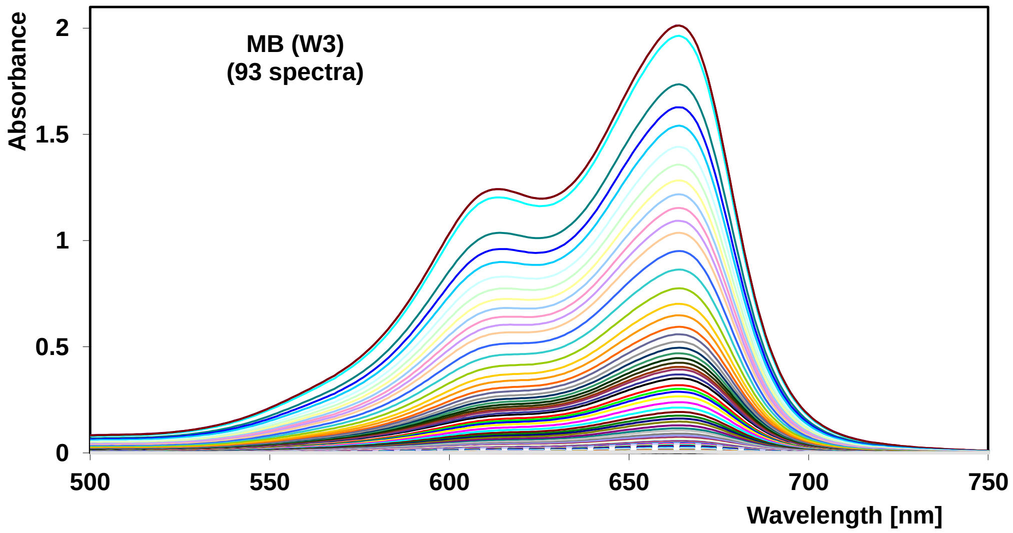

Figure 1. Exemplary absorption spectra in the Vis range measured during the adsorption process for the MB (W3) system.

Table 2. Comparison of the parameters of the FOE, SOE, MOE and multi-exponential kinetic equations for the studied systems.

Kinetic system	fit	i	f_i	log k_i	$t_{0.5}$ [min]	u_{eq}	SD(c/c_0)[%]	1-R^2
MB (RIAA)	FOE	2	0	-2.92	575	0.980	1.16	$1.02 \cdot 10^{-3}$
	SOE	2	1	-2.66	461	1	4.97	$2.21 \cdot 10^{-2}$
	MOE	2	0.367	-3.06	565	0.996	0.61	$2.84 \cdot 10^{-4}$
	m-exp	--	--	-2.92 (av.)	572 (av.)	1	0.18	$2.42 \cdot 10^{-5}$
		1	0.08	-2.05	78			
		2	0.91	-2.97	642			
		3	0.01	-6.07	805509			
MB(W1)	FOE	2	0	-2.41	176	0.975	1.72	$2.63 \cdot 10^{-3}$
	SOE	2	1	-2.11	129	1	4.83	$2.42 \cdot 10^{-2}$
	MOE	2	0.526	-2.63	167	1	0.67	$3.89 \cdot 10^{-4}$
	m-exp	--	--	-2.36 (av.)	158 (av.)	0.998	0.28	$6.28 \cdot 10^{-5}$
		1	0.05	-0.35	2			
		2	0.31	-2.08	84			
		3	0.64	-2.57	257			
MB(W2)	FOE	2	0	-1.95	61	0.953	0.90	$4.10 \cdot 10^{-2}$
	SOE	2	1	-1.64	43	1	3.25	$1.67 \cdot 10^{-2}$
	MOE	2	0.981	-3.37	45	1	3.23	$1.60 \cdot 10^{-2}$
	m-exp	--	--	-1.52 (av.)	23 (av.)	0.987	0.72	$7.48 \cdot 10^{-4}$
		1	0.15	-0.25	1			
		2	0.34	-1.06	8			
		3	0.51	-2.32	144			
MB(W3)	FOE	2	0	-1.39	17	0.980	2.44	$1.08 \cdot 10^{-2}$
	SOE	2	1	-1.01	10	1	2.71	$1.45 \cdot 10^{-2}$
	MOE	2	0.780	-1.82	13	0.996	0.75	$9.93 \cdot 10^{-4}$
	m-exp	--	--	-1.28 (av.)	13 (av.)	0.998	0.59	$6.81 \cdot 10^{-4}$

		1	0.54	-1.02	7			
		2	0.45	-1.66	32			
		3	0.01	-2.64	301			
MO (RIAA)	FOE	2	0	-2.90	556	0.985	0.83	$5.55\cdot10^{-4}$
	SOE	2	1	-2.64	434	1	5.40	$2.77\cdot10^{-2}$
	MOE	2	0.282	-3.01	550	1	0.30	$7.38\cdot10^{-5}$
	m-exp	--	--	-2.90 (av.)	549 (av.)	1	0.25	$4.96\cdot10^{-5}$
		1	0.01	-1.76	40			
		2	0.16	-2.56	252			
		3	0.82	-2.98	665			
MO(W1)	FOE	2	0	-2.79	424	0.904	3.41	$1.39\cdot10^{-2}$
	SOE	2	1	-2.60	398	1	1.66	$3.39\cdot10^{-3}$
	MOE	2	0.931	-3.80	419	1	1.36	$2.18\cdot10^{-3}$
	m-exp	--	--	-2.67 (av.)	326 (av.)	0.963	0.45	$2.29\cdot10^{-4}$
		1	0.08	-0.66	3			
		2	0.31	-2.24	120			
		3	0.60	-3.06	789			
MO(W2)	FOE	2	0	-2.15	97	0.948	5.16	$5.84\cdot10^{-2}$
	SOE	2	1	-1.76	58	1	3.75	$3.04\cdot10^{-2}$
	MOE	2	0.988	-3.71	60	1	3.76	$2.99\cdot10^{-2}$
	m-exp	--	--	-1.52 (av.)	23 (av.)	0.983	0.80	$1.37\cdot10^{-3}$
		1	0.31	-0.52	2			
		2	0.23	-1.35	15			
		3	0.46	-2.40	176			
MR (RIAA)	FOE	2	0	-3.04	768	0.950	2.24	$4.08\cdot10^{-3}$
	SOE	2	1	-2.82	667	1	3.18	$9.14\cdot10^{-3}$
	MOE	2	0.707	-3.47	762	1	0.67	$3.67\cdot10^{-4}$
	m-exp	--	--	-3.03 (av.)	745 (av.)	0.990	0.18	$2.60\cdot10^{-5}$
		1	0.06	-1.90	54			
		2	0.34	-2.75	386			
		3	0.60	-3.29	1348			
MR(W1)	FOE	2	0	-2.72	364	0.954	3.13	$8.34\cdot10^{-3}$
	SOE	2	1	-2.45	283	1	2.55	$5.93\cdot10^{-3}$
	MOE	2	0.822	-3.29	323	0.997	1.17	$1.15\cdot10^{-3}$
	m-exp	--	--	-2.63 (av.)	294 (av.)	0.986	0.29	$6.71\cdot10^{-5}$
		1	0.08	-1.23	12			
		2	0.32	-2.28	132			
		3	0.59	-2.96	637			
MR(W2)	FOE	2	0	-1.83	47	0.949	4.35	$4.33\cdot10^{-2}$
	SOE	2	1	-1.48	30	1	1.92	$8.32\cdot10^{-3}$
	MOE	2	0.984	-3.30	31	1	1.89	$7.87\cdot10^{-3}$
	m-exp	--	--	-1.46 (av.)	20 (av.)	0.986	0.57	$6.79\cdot10^{-4}$
		1	0.15	-0.05	1			
		2	0.41	-1.20	11			
		3	0.44	-2.16	100			
CV (RIAA)	FOE	2	0	-3.20	1104	0.800	2.70	$1.17\cdot10^{-2}$
	SOE	2	1	-3.18	1516	0.996	1.54	$3.78\cdot10^{-3}$
	MOE	2	0.997	-5.71	1511	0.995	1.54	$3.78\cdot10^{-3}$
	m-exp	--	--	-3.34 (av.)	1516 (av.)	1	0.22	$7.79\cdot10^{-5}$

		1	0.07	-1.97	64			
		2	0.28	-2.84	479			
		3	0.65	-3.67	3275			
CV(W1)	FOE	2	0	-2.84	479	0.908	2.57	$7.78\cdot10^{-3}$
	SOE	2	1	-2.64	434	1	2.45	$7.51\cdot10^{-3}$
	MOE	2	0.795	-3.42	494	1	1.25	$1.82\cdot10^{-3}$
	m-exp	--	--	-2.79 (av.)	423 (av.)	0.971	0.17	$3.23\cdot10^{-5}$
		1	0.07	-1.20	11			
		2	0.23	-2.28	131			
		3	0.70	-3.04	766			
CV(W2)	FOE	2	0	-2.54	238	0.899	4.86	$3.79\cdot10^{-2}$
	SOE	2	1	-2.32	210	0.992	2.85	$1.27\cdot10^{-2}$
	MOE	2	0.999	-5.19	210	0.991	2.86	$1.27\cdot10^{-2}$
	m-exp	--	--	-2.22 (av.)	115 (av.)	0.971	0.46	$3.39\cdot10^{-4}$
		1	0.17	-0.65	3			
		2	0.33	-1.85	49			
		3	0.51	-2.89	540			

Thermal Degradation of the Systems

In order to study thermal degradation of the dye/activated carbon system the measurements applying thermal analysis were made. In Figure S3(a–c) the TG, DTG and DSC curves measured for methylene blue, the pure carbon W3 and W3 loaded with dye are presented.

Analysing the process of thermal degradation of methylene blue (Figure S3a) one can identify many stages on the DTG curve. This is a result of its complicated molecular structure – the presence of two benzene rings joined by sulphur and nitrogen atoms, and side dimethylamino groups. In the temperature range of 25–160 °C the endothermic peak corresponding to the removal of the physically bound, hygroscopic water is observed. The hygroscopicity of methylene blue is related to the hydrophilic character of molecule (Log P= -0.9) responsible for hydrogen bonds between water and sulphur and/or nitrogen atoms. The subsequent exothermic events up to 600 °C, with the predominant peak with the maximum rate at 444 °C, indicate oxidation and pyrolysis processes of the organic dye [76].

The DTG curve for the mesoporous carbon W3 (Figure S3b) shows a well defined exothermic peak with the total weight loss of 91 wt.% of sample mass. In the temperature range smaller than 450 °C successive desorption of thermally unstable oxygen complexes (carboxylic groups, lactones or lactols) bound to the carbonaceous surface occurs. It is well visible as an asymmetric peak on the DSC curve. In the temperature range 450–650 °C the exothermic processes of oxidation of the more stable oxygen species (with a complex structure) and pyrolysis of solid bulk take place. This stage is visible as the single peak with the minimum at 490 °C. Possible oxygen complexes which undergo degradation are as follows: phenolic, hydroquinonic, carbonylic, quinonic groups or ethers.

In the case of carbonaceous material loaded with dye (Figure S3c), two peaks on the DTG curve are observed. The first one with the minimum at 60 °C corresponds to removal of the weakly bound water whereas the subsequent one, with a bimodal character and the minimum at 421 and 474 °C can be attributed to the thermal decomposition of adsorbate and adsorbent. In comparison to the native material the thermal profile shows a shift towards lower temperature values (from 490 to 474 °C). Therefore, one can state that the dye adsorption on W3 results in change of the degradation temperature range for the carbonaceous material.

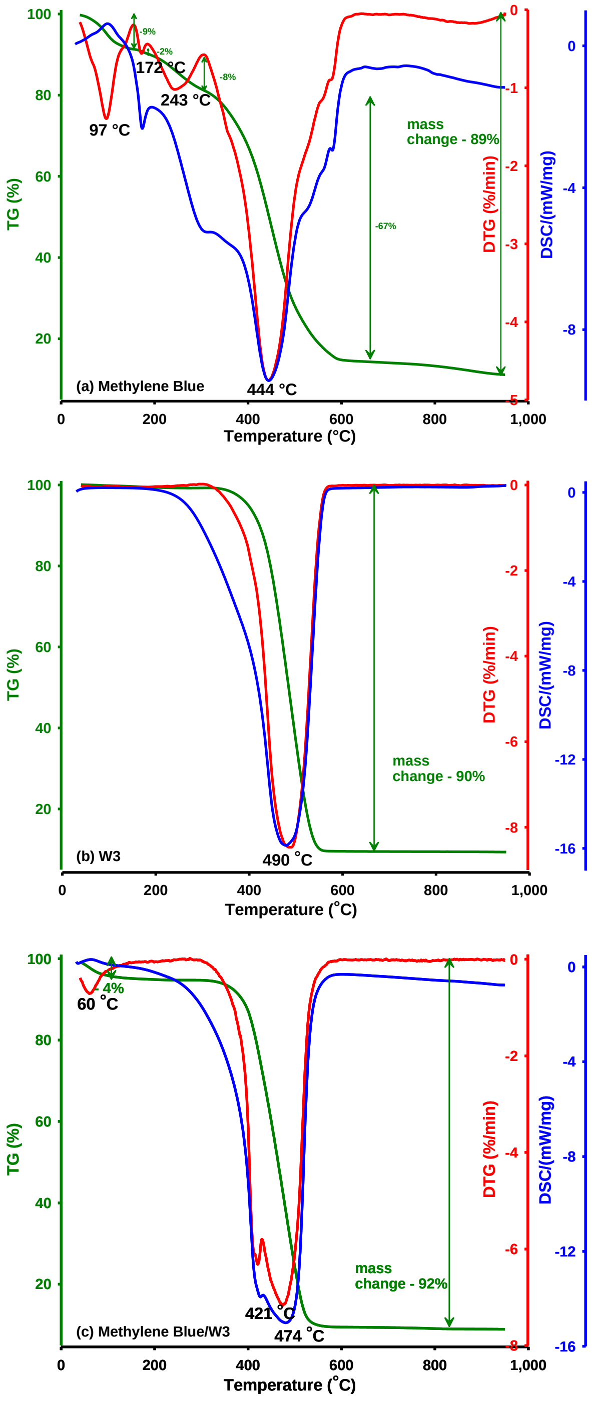

Figure 2. Comparison of the TG, DTG and DSC curves measured for methylene blue (a), W3 (b) and methylene blue /W3 systems (c).

Volatile products released during the thermal degradation of methylene blue, carbonaceous material W3 and W3 loaded with dye were analyzed using the FTIR technique as shown in Figure S4. During the pyrolysis of all samples the most characteristic infrared bands centered at 2360, 2310 cm^{-1} and 669 cm^{-1} may be attributed to stretching and deformation vibrations of CO_2, respectively. CO_2 was generated in the thermal destruction of multiple benzene rings of dye, surface oxygen species and solid bulk of W3. At the temperatures above 243 °C some of these processes proceed with simultaneous release of CO with characteristic bands in the range of 2000–2500 cm^{-1}. Moreover, the wide absorption bands from 3500 cm^{-1} to 4000 cm^{-1} and additionally at 1756 cm^{-1} correspond to the O–H stretching vibrations and symmetric bending of water molecules in the vapor phase. The

peaks at 1537 cm^{-1} and 1542 cm^{-1} indicate the stretching vibrations of C=C bonds in the aromatic dye ring or the carbon cyclic structure.

In the case of the spectra for pure dye and dye/W3 samples one can observe the signal recorded for SO_2 as a band at 1370 cm^{-1} (asymmetric stretching vibrations) which come from sulphur atoms in the dye molecules. There is also a peak at 714 cm^{-1} on the spectra for both samples indicating the presence of HCN in the gaseous phase. In turn, the absorption bands at 2896 cm^{-1} and 2972 cm^{-1} corresponding to the CH stretching vibrations of methyl groups CH_3 are well defined only for the dye sample. However, this signal is only recorded at 172 and 243 °C, and for these temperatures the spectra for dye/W34 are not shown.

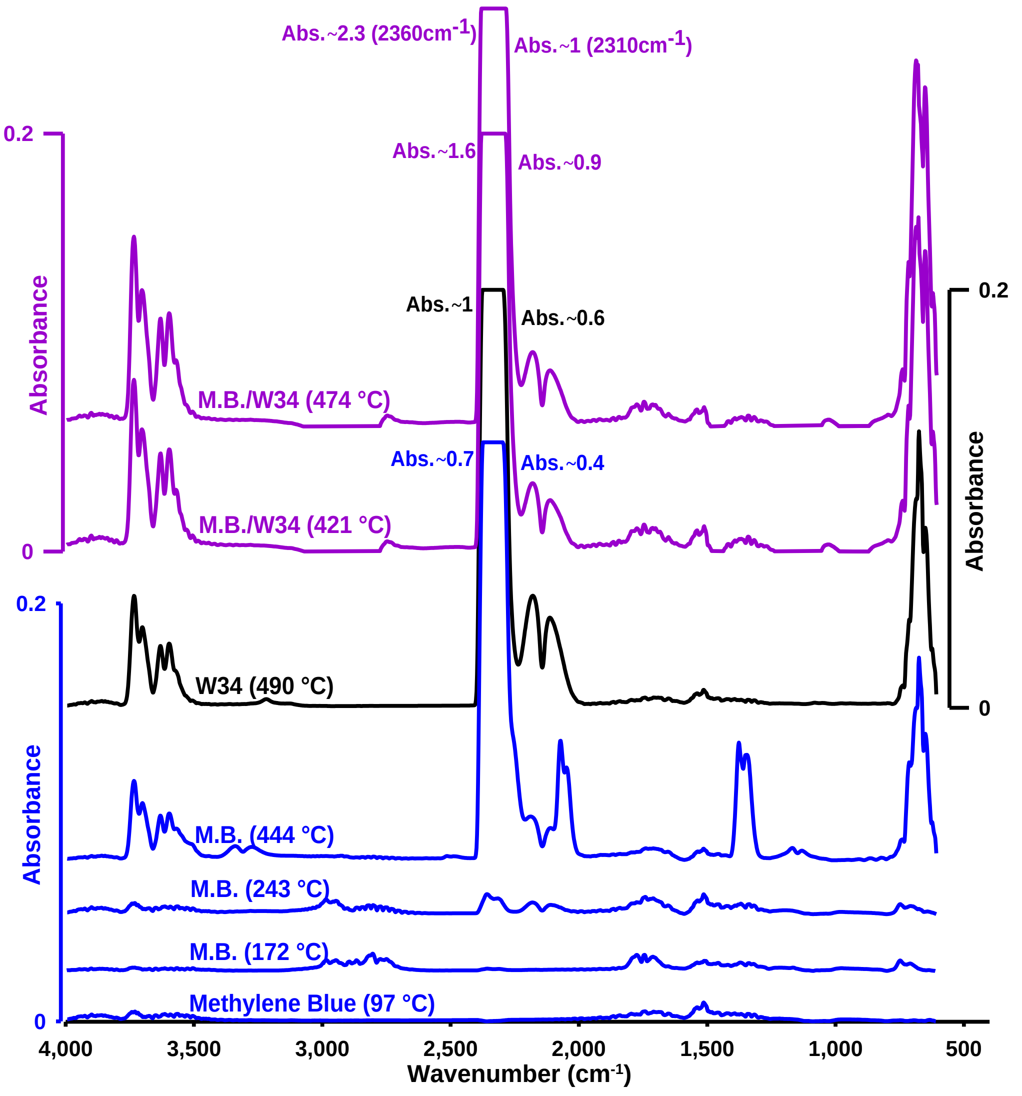

Figure 3. FTIR spectra of gas products of pyrolysis of methylene blue, W3 and methylene blue /W3 system at the temperatures corresponding to the process rate maxima.

Mesoporous carbon preparation

Table 3. Physicochemical properties of the amphiphilic triblock copolymers used in the silica synthesis.

Pluronic	Chemical formula	Molecular weight [g/mol]	Molar mass of PEO block
PE9400	$(EO)_{21}(PO)_{47}(EO)_{21}$	4600	2750
PE10500	$(EO)_{37}(PO)_{56}(EO)_{37}$	6500	3250

Table 4. Parameters of the synthesis of mesoporous silicas and carbons.

Carbon	Pluronic	Temperature /time of silica ageing process	Temperature /time of silica calcination	Carbon precursor	Temperature /time of carbonization
W1	PE9400	120 °C/86 h	500 °C/6 h	saccharose	800 °C/6 h
W2	PE9400	70 °C/24 h	500 °C/6 h	glucose	800 °C/6 h
W3	PE10500	120 °C/24 h	500 °C/6 h	glucose	800 °C/6 h

Adsorbates

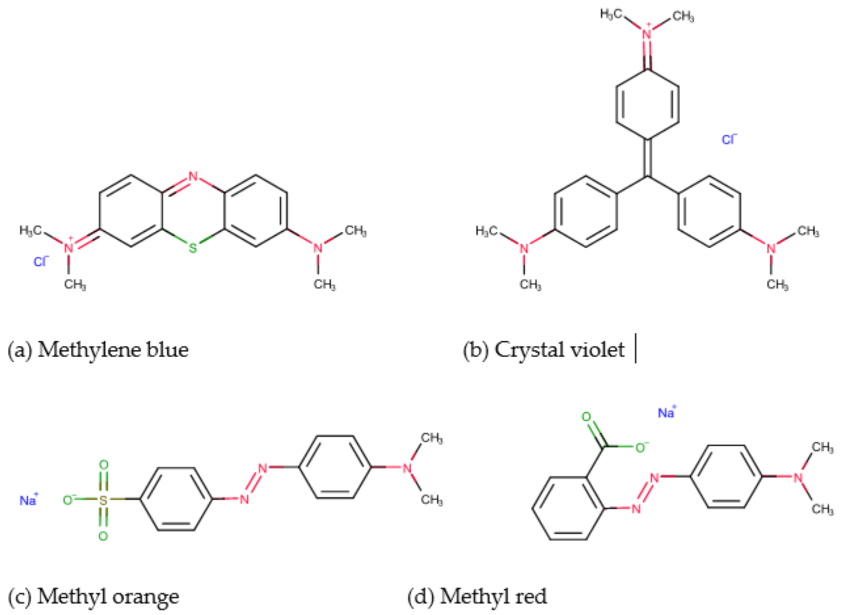

Figure 4. Chemical structure of the dyes: (a) methylene blue; (b) crystal violet; (c) methyl orange; (d) methyl red.

Adsorption Experiment

Adsorption Equilibrium

Table 5. Generalized Langmuir isotherm equation (GL) and its simplified forms.

Isotherm (code)	Equation	Isotherm (code)	Equation	m	n
Generalized Langmuir (GL)	$\theta = \left[\frac{(Kc_{eq})^n}{1+(Kc_{eq})^n}\right]^m$	Langmuir-Freundlich (LF)	$\theta = \frac{(Kc_{eq})^m}{1+(Kc_{eq})^m}$	$\in (0,1$ m=n	$\in (0,1$ m=n
		Generalized Freundlich (GF)	$\theta = \left[\frac{Kc_{eq}}{1+Kc_{eq}}\right]^m$	$\in (0,1$	1
		Tóth (T)	$\theta = \frac{Kc_{eq}}{[1+(Kc_{eq})^n]^{1/}}$	1	$\in (0,1$
		Langmuir (L)	$\theta = \frac{Kc_{eq}}{1+Kc_{eq}}$	1	1

where: θ is the global (overall) adsorption isotherm (overall coverage), m and n are the heterogeneity parameters characterizing a shape (width and asymmetry) of the adsorption energy

distribution function; *K* describes the adsorption equilibrium constant characterizing a position of the distribution function on an energy axis.

Adsorption Kinetics

Table 6. Various kinetic equations applied in the study.

Kinetic equation (code)	General form	Linear form
First-order (FOE)	$\frac{dc}{dt} = -k_1(c - c_{eq})$	$\ln(c - c_{eq}) = \ln(c_o - c_{eq}) - k_1 t$
Pseudo-first order (PFOE)	$\frac{da}{dt} = k_1(a - a_{eq})$	$\ln(a_{eq} - a) = \ln a_{eq} - k_1 t$
Second-order (SOE)	$\frac{dc}{dt} = -k_{2c}(c - c_{eq})^2$	—
Pseudo-second order (PSOE)	$\frac{da}{dt} = k_{2a}(a_{eq} - a)^2$	$\frac{t}{a} = \frac{1}{k_2 a_{eq}^2} + \frac{t}{a_{eq}}$ *or* $a = a_{eq} - \frac{1}{k_{2a} a_{eq}} \frac{a}{t}$
Mixed 1.2-order (MOE)	$F = \frac{a}{a_{eq}} = \frac{1 - exp(-k_1 t)}{1 - f_2 exp(-k_1 t)}$	$ln\left(\frac{1 - F}{1 - f_2 F}\right) = -k_1 t$
Multi-exponential (m-exp)	$c = (c_o - c_{eq}) \sum_{i=1}^{n} f_i \exp(-k_i t) + c_{eq}$	—
	$a = a_{eq}[1 - \sum_{i=1}^{n} f_i \exp(-k_i t)]$	—

where: a_{eq} and c_{eq} are the equilibrium adsorption and concentration, c_o is the initial concentration, *c* and *a* are the temporary concentration and adsorption, *V* is the solution volume, *m* is the adsorbent mass, *k* is the kinetic rate coefficient and *t* is time, *F* is the adsorption progress, $f_2<1$ is normalized share of second-order term in the overall rate dependence.
